# Amine groups alter product selectivity and rate of catalytic hydride transfer reactions†

**DOI:** 10.1039/d4sc07359b

**Published:** 2025-02-13

**Authors:** Santanu Pattanayak, Rachel E. Siegel, Yiming Liu, James C. Fettinger, Louise A. Berben

**Affiliations:** a Department of Chemistry, University of California Davis CA 95616 USA laberben@ucdavis.edu

## Abstract

Primary amines are common functional groups in the reaction environment surrounding an (electro)catalyst, and this includes catalysts ranging from metalloenzymes surrounded by amino acids, to electrocatalysts operating in amine industrial sorbents for CO_2_ capture and conversion. This report explores the behavior of amine functional groups at the surface of an electrocatalyst. The possible effects of those amine secondary coordination sphere (SCS) groups on a CO_2_ electro-reduction mechanism include stabilization of intermediates and positioning substrate near the active site. Two different clusters were synthesized: [PPN][Fe_4_N(CO)_11_(Ph_2_PCH_2_CH_2_NH_2_)] (PPN-1) has one amine, and [PPN][Fe_4_N(CO)_10_(Ph_2_PCH_2_CH_2_NH_2_)_2_] (PPN-2) has two covalently appended amine functional groups (PPN: bis(triphenylphosphine)iminium). Infra-red spectroscopic studies show a direct reaction of each cluster with CO_2_ to afford an SCS carbamate functional group, and cyclic voltammetry investigations reveal a variety of roles for the amine SCS groups in the mechanism of catalyst hydride formation and hydride transfer (HT) to CO_2_. The most prominent effect of the amine functional group is stabilization of the intermediate hydride to lower formate yield. With PPN-1, these combined effects serve to shut down HT to CO_2_. With PPN-2, the combined effects result in some loss of selectivity, so that formate and H_2_ mixtures (6 : 1) are obtained.

## Abstract 2

An electrocatalyst is modified with amines at the surface, to model a common reaction environment. The most prominent effect of the amines is hydrogen bonding with the catalyst-hydride intermediate, and alterations to product selectivity.

## Graphical abstract

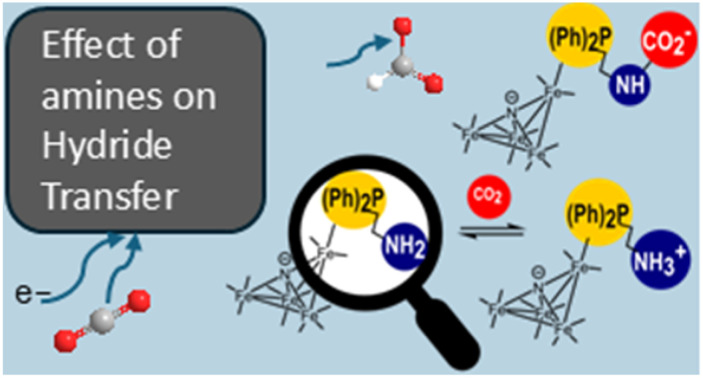

## Introduction

Specific interactions of functional groups near the surface of heterogeneous electrocatalysts or near the active site of a molecular or biological catalyst,^1,2^ are often responsible for the observed catalysis outcomes such as reaction rate, product selectivity, and overpotential, amongst others.^3,4^ As specific examples in biology, carbonic anhydrase and hydrogenase activity have well-documented mechanisms. Carbonic anhydrase is an enzyme that catalyzes the reversible conversion of CO_2_ and water into bicarbonate and protons.^5^ In the active site of carbonic anhydrase, hydrogen bond (H-bond) donors are known to modulate p*K*_a_ so that reversible proton transfer (PT) to CO_2_ is possible at fast rates.^6^ The [FeFe]-hydrogenase enzyme reduces H^+^ into H_2_*via* a hydride transfer (HT) mechanism. In that mechanism, it has been proposed that amine functional groups in amino acids are involved both in the maturation process as well as catalysis, and that the role of amines is likely as a Lewis base.^7^ It has also been proposed that amines are structurally important to [FeFe]-hydrogenases, where H-bonding between the hydride of Hhyd and the ADT-NH is thought to stabilize the structure.^8^

In biology, amine functional groups can have several roles, including as H-bond donors, or as Lewis basic sites that aid in proton transport. Similar roles are known in non-biological catalytic systems. It would be very useful to learn more about the multitude of roles that secondary coordination sphere (SCS) primary amine functional groups can play in the atomic level details of catalytic CO_2_ reduction mechanisms. Specific examples of amine functional groups in electrocatalysis include a study on the role of amines at the surface of Ag and Cu electrocatalyst which promote CO_2_ reduction. In that instance, multiple roles including H-bond stabilization of CO_2_ and tuning the reorganization energy of the surrounding water were proposed.^9–11^ Studies with molecular catalysts which probe the role of amine functional groups on CO_2_ reduction catalysis include work where amines stabilize intermediate Fe–CO interactions so that further reduction to CH_4_ can occur using an iron porphyrin complex,^12^ and alternatively where amines stabilize a Co-carboxylate intermediate along a pathway to enhanced CO formation rate.^13^ Amine functional groups have also been used as a proton shuttle to favor formate over CO production, from CO_2_ reduction.^14,15^

**Chart 1:**
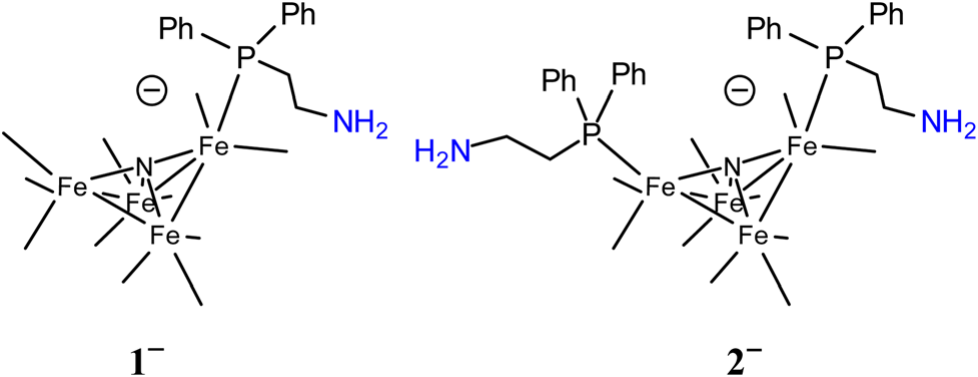
Line drawings of 1^−^ (left) and 2^−^ (right). CO ligands are omitted. Atom numbering scheme is in ESI (Chart S1).†

Molecular models can provide mechanistic insights into possible roles that primary amine functional groups can have in CO_2_ reduction catalysis. In this report, we focused on installation of a primary amine SCS group at the surface of a small cluster electrocatalyst, [Fe_4_N(CO)_12_]^–^ (Chart 1).^16^ This electrocatalyst was chosen because we have previously demonstrated selective formation of formate using [Fe_4_N(CO)_12_]^–^: and that reaction mechanism involves a key hydride intermediate [H–Fe_4_N(CO)_12_]^–^, that selectively transfers H^−^ to CO_2_ to afford formate. In the work reported herein, multiple roles for the surface amine functional groups have been uncovered, including direct reaction with CO_2_ to afford carbamate anion and as a Lewis basic site to accept protons and yield ammonium which then serves as proton sources for hydride formation or as H-bond stabilization of the catalytic intermediate hydride [HFe_4_N(CO)_12_]^–^. The sometimes cooperative and sometimes competing effects of these various roles for surface amine groups on [Fe_4_N(CO)_11_(PPh_2_(CH_2_)_2_NH_2_)]^–^ (1^−^) and [Fe_4_N(CO)_10_(PPh_2_(CH_2_)_2_NH_2_)_2_]^–^ (2^−^) are discussed below. Optimization of the catalytic reaction conditions ultimately employs the amine groups on 2^−^ for CO_2_ capture, and the observed rate constant for formate production is 7.3 s^−1^. This rate constant is of the same order of magnitude reported for [Fe_4_N(CO)_12_]^–^ to make formate,^17^ and that result suggests that dissolved CO_2_ is the source of substrate.

## Results and discussion

### Synthesis of amine-substituted catalysts, 1^−^ and 2^−^

The SCS amine tagged phosphine substituted cluster Et_4_N[Fe_4_N(CO)_11_(Ph_2_P(CH_2_)_2_NH_2_)] (Et_4_N-1) was synthesized in quantitative yield by addition of one equivalent of Ph_2_P(CH_2_)_2_NH_2_ to [Na(diglyme)_2_][Fe_4_N(CO)_12_], followed by heating in THF at 60 °C for 12 h, before Et_4_NCl was used in a salt metathesis reaction that afforded Et_4_N-1. The detailed synthetic procedures are given in the ESI†, and (Et_4_N)^+^ is the cation tetraethylammonium. Synthesis of doubly substituted Et_4_N[Fe_4_N(CO)_10_(Ph_2_P(CH_2_)_2_NH_2_)_2_] (Et_4_N-2) was achieved in 65% yield; 3.5 equivalents of Ph_2_P(CH_2_)_2_NH_2_ was heated at reflux with [Na(diglyme)_2_][Fe_4_N(CO)_12_] in THF/toluene (1 : 3 v/v) for 24 h, followed by salt metathesis reaction with Et_4_NCl and workup. Following identical salt metathesis procedures with PPNCl, we were also able to isolate PPN-1 and PPN-2 (PPN is the non-coordinating cation bis(triphenylphosphine)iminium). Throughout the manuscript, we henceforth refer to Et_4_N-1 or PPN-1 and Et_4_N-2 or PPN-2 as 1^−^ and 2^−^, respectively.

Each of the clusters, 1^−^ and 2^−^, was characterized by ^1^H, ^13^C, and ^31^P NMR, and IR spectroscopy (Fig. S1–S7†); and the ^31^P NMR spectra each show a single sharp resonance approximately 70 ppm downfield from the free phosphine ligand (Ph_2_PCH_2_CH_2_NH_2_ shows ^31^P signal at −21 ppm). Analogous phosphine-substituted compounds such as [Fe_4_N(CO)_11_(Ph_2_PCH_2_CH_2_OH)]^–^ also have ∼70 ppm downfield shift in the ^31^P NMR resonances. Combustion analysis was performed to confirm bulk purity of each compound. The CO absorption bands (*ν*_CO_) in the IR spectra of 1^−^ and 2^−^ are slightly shifted to lower energy, relative to those in the unsubstituted cluster [Fe_4_N(CO)_12_]^–^ because the phosphine ligands are more weakly π-accepting: for [Fe_4_N(CO)_12_]^–^, the bands are at 2019 and 1989 cm^−1^; for 1^−^, they are at 1985 and 1970 cm^−1^, and for 2^−^, they are at 1959 and 1943 cm^−1^ (Fig. S4†).

**Fig. 1:**
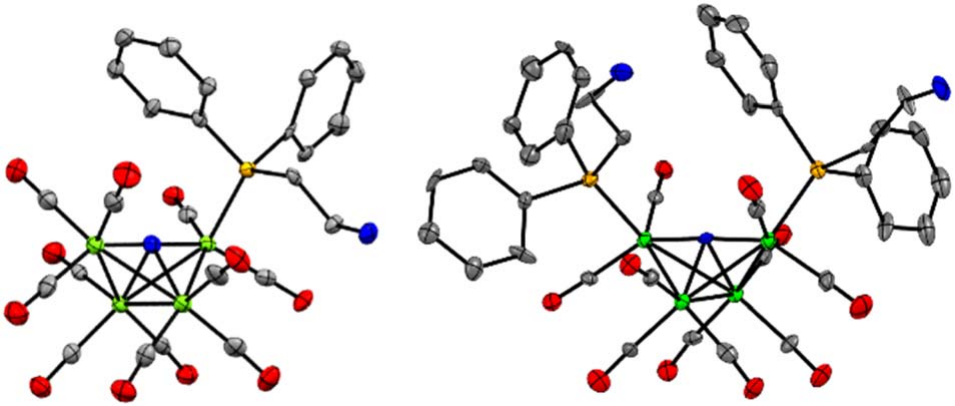
Solid state structures of (left) 1^−^ in Et_4_N-1·THF, ^18^ and (right) 2^−^ in PPN-2. ^19^ Gray, blue, green, orange ellipsoids represent C, N, Fe, O and P atoms, respectively. Ellipsoids are shown at 50%; counter cations (Et_4_N^+^ and PPN^+^), solvates, and H atoms are omitted for clarity.

X-ray diffraction quality crystals for Et_4_N-1 ^18^ and PPN-2 ^19^ were grown out of layered THF-hexane and saturated toluene solutions, respectively, kept at −16 °C over 3 or 8 days, respectively (Fig. 1 and Tables S1, S2†). Comparison of Fe–P bond length in Et_4_N-1 and PPN-2 reveals that one of the Fe–P bonds in 2^−^ is slightly elongated (2.210(5) and 2.2070(6) Å), relative to the shorter Fe–P bond in 1^−^, which is 2.2066(10) Å. This is likely a steric effect: we have previously noted that Fe–P bond lengths correlate with the size (Tolman cone angle)^20^ of a phosphine ligand.^21–23^ The Fe(1)–P bond distance (2.2028(6) Å) in similar cluster [Fe_4_N(CO)_11_(Ph_2_PCH_2_CH_2_OH)]^–^ is also found to be very close to that of 1^−^. Replacement of CO by phosphine ligand has a small impact on the Fe–N bond lengths in both 1^−^ and 2^−^. The Fe–N (Fe1–N, Fe4–N, Fe2–N, and Fe3–N) bond lengths in 1^−^ are 1.780(3), 1.771(3), 1.907(3), and 1.915(3) Å, respectively; and those in 2^−^ are 1.7857(16), 1.7922(16), 1.9235(15), and 1.9270(15) Å (see ESI for Fe numbering scheme; Chart S1†). The small variations in the structural parameters of [Fe_4_N(CO)_12_]^–^, 1^−^, and 2^−^, reflect the delocalized bonding which distributes changes in electron density across the metal core.

### Electrochemical experiments

**Fig. 2:**
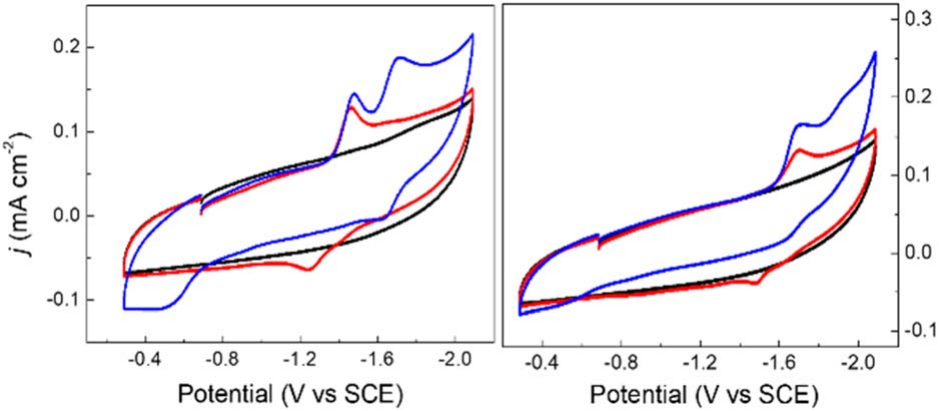
Cyclic voltammograms of 0.1 mM PPN-1 (left) and PPN-2 (right); in 0.1 M Bu_4_NBF_4_ MeCN, under 1 atm of N_2_ (red) or CO_2_ (blue). Blank recorded under N_2_ (black). GC electrode; scan rate: 0.1 V s^−1^.

Cyclic voltammograms (CVs) of 0.1 mM solutions of 1^−^ and 2^−^ were first measured in anhydrous 0.1 M Bu_4_NBF_4_ MeCN solution under 1 atm N_2_ (Fig. 2). One reduction event with cathodic peak potential (*E*_p,c_) at −1.46 V and anodic peak potential (*E*_p,a_) at −1.22 V *vs.* SCE was observed for 1^−^ (Δ*E*_p_ = 240 mV). Cyclic voltammograms of other mono-substituted clusters, such as [Fe_4_N(CO)_11_(PPh_3_)]^–^ and [Fe_4_N(CO)_11_(Ph_2_PCH_2_CH_2_OH)]^–^, showed *E*_p,c_ = −1.49 V which is consistent with this result. We have previously shown that differential pulse voltammetry (DPV) gives a good estimation of *E*_½_ when cluster cyclic voltammograms are not fully reversible,^24^ and in this case differential pulse voltammetry suggests that *E*_½_(1^−/2−^) = −1.43 V (Fig. S8†). The electrochemical response of 0.1 mM PPN-2 in 0.1 M Bu_4_NBF_4_ MeCN (Fig. 2, right) under 1 atm N_2_ shows an irreversible reduction event at −1.67 V *vs.* SCE, and the differential pulse voltammetry experiment indicates that *E*_½_(2^−/2−^) = −1.65 V. The cathodic shift of 220 mV, relative to 1^−^, is consistent with the more electron-rich cluster core after substitution with two phosphine ligands. A linear relationship has previously been demonstrated between *ν*_CO_ and *E*_p_ for [Fe_4_N(CO)_12_]^–^ and its substituted analogues.^23,25^ Both 1^−^ and 2^−^ fall on this line as is expected.

For both 1^−^ and 2^−^, an analysis of variable scan rate cyclic voltammograms showed a linear variation of peak current density (*j*_p_) with scan rate (*υ*^1/2^), although neither compound is rigorously electrochemically irreversible (Fig. S9†). The differential pulse voltammetry experiments with 1^−^ and 2^−^, were used to support the assignment of each reduction event under N_2_ as a one-electron transfer (Fig. S8†).

### Reactivity of PPN-1 and PPN-2 with CO_2_

**Scheme 1:**
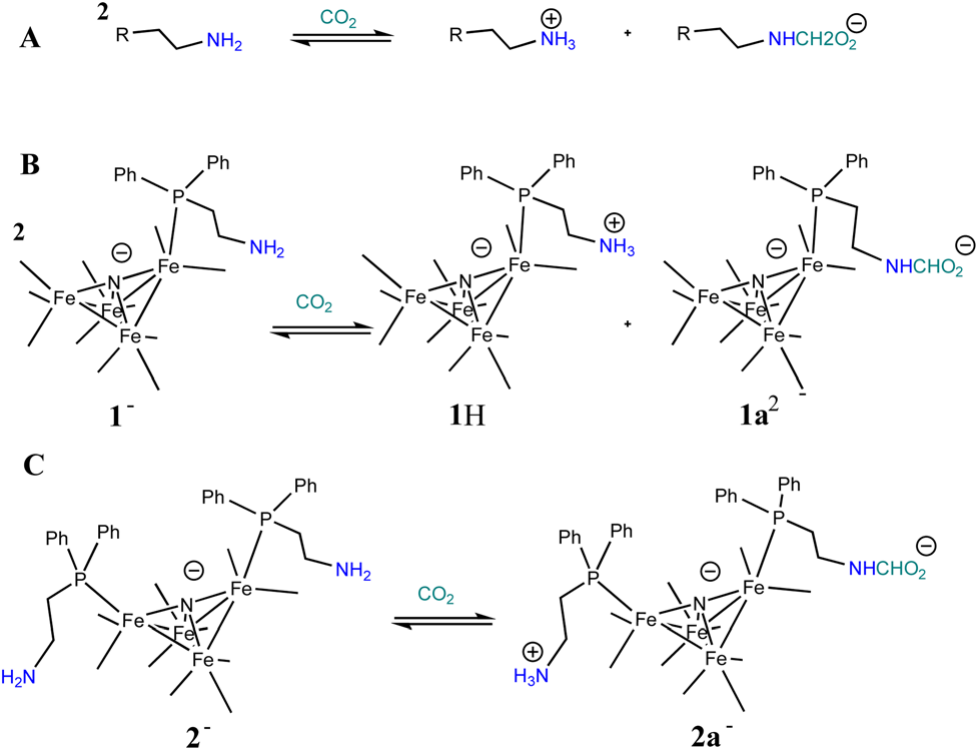
General known reaction of amines with CO_2_ (A); and proposed reaction of 1^−^ with CO_2_ (B), and of 2^−^ with CO_2_ (C).

**Fig. 3:**
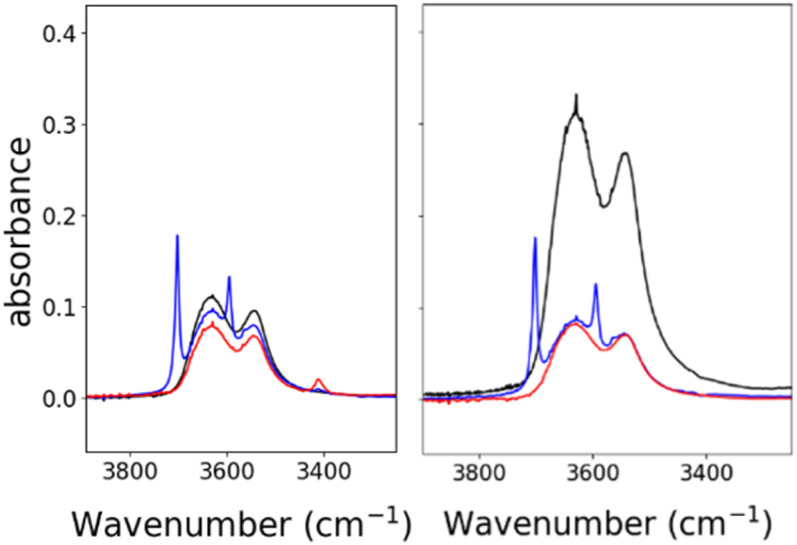
IR spectra showing N–H absorption bands for: 0.7 mM 1^−^ (left) and 2^−^ (right), both in MeCN. Under 1 atm of N_2_ (black), under 1 atm CO_2_ (blue), and with 0.7 mM of benzoic acid under 1 atm N_2_ (red). Full spectral ranges are shown in Fig. S10.†

Based on the known chemistry of CO_2_ reactions with amines (Scheme 1A),^26^ we expected that reaction of 1^−^ with CO_2_ might afford (PPN)_2_[Fe_4_N(CO)_11_(Ph_2_P(CH_2_)_2_NH_3_^+^)][Fe_4_N(CO)_11_(Ph_2_P(CH_2_)_2_NHCO_2_^−^)], which we denote as PPN_2_(1H^+^)(1a^2−^) (Scheme 1B). A reaction of 1^−^ with CO_2_ might afford PPN[Fe_4_N(CO)_10_(Ph_2_P(CH_2_)_2_NH_3_^+^)(Ph_2_P(CH_2_)_2_NHCO_2_^−^)], or PPN(2a^−^) (Scheme 1C). We monitored reactions of 1^−^ and of 2^−^ with CO_2_ in MeCN using infra-red spectroscopy. There was no change of the *ν*_CO_ absorption bands over 5 h, and this suggests that the cluster core electronic properties are unchanged (Fig. S10†). The IR spectra collected under N_2_ and CO_2_ do show changes in the region of 3300–3800 cm^−1^, and these are consistent with changes in the N–H absorption bands due to carbamate formation (Fig. 3). Under 1 atm CO_2_, sharp absorption bands appear at 3701 and 3594 cm^−1^ in the spectra of both 1^−^ and 2^−^, and these are consistent with carbamate formation.^27,28^ To check carbamate under our own conditions, an IR spectrum of dimethylammonium dimethylcarbamate was collected, and shows sharp bands at 3700 and 3595 cm^−1^ (Fig. S10,† right). For comparison, we also collected the IR spectra of 1^−^ and 2^−^ under N_2_ with 1 equivalent of added benzoic acid (BnCOOH, p*K*_a_ = 21.5 in MeCN),^29^ since the BnCOOH should protonate the amine functional groups to afford 1H and 2H, respectively (Scheme 1). These spectra showed very little change relative to those of 1^−^ and 2^−^, and this confirms that the sharp bands observed under 1 atm CO_2_ are not associated with amine protonation (Fig. S10†). Previous studies have observed only small amounts of free amine following reaction with CO_2_ in MeCN.^24^ Our IR analysis is in agreement with that result, although precise concentrations cannot be calculated from the acquired spectra. We therefore estimate the equilibrium constant (*K*_eq_) for CO_2_ binding as described in Scheme 1A and B to be high, with *K*_eq_ > 10.

We will first discuss the electrochemical data associated with the CO_2_ reactivity of PPN-1. As mentioned in the previous paragraph, there is no change to the *ν*_CO_ absorption bands for 1^−^ upon reaction with CO_2_; this suggests that the cluster core electronic properties are unchanged, and therefore, that the *E*_½_ values for 1H^0/–^ and 1a^2−/3−^ should be equivalent to *E*_½_(1^−/2−^). Cyclic voltammetry measurements were performed in 0.1 M Bu_4_NBF_4_ MeCN saturated with CO_2_ (Fig. 2, blue). Under these conditions, the quasi-reversible cyclic voltammogram of 1^−^ under 1 atm N_2_ becomes an irreversible event at −1.43 V, and a new reversible wave at more cathodic potential (*E*_1/2_ = −1.62 V *vs.* SCE, Δ*E*_p_ = 100 mV) was observed (Fig. 2, left). No change in the irreversibility was observed when the scan rate is faster (Fig. S11†). Since the redox event for 1H^0/–^ and 1a^2−/3−^ is irreversible under CO_2_, we cannot use this to calculate *K*_b_ for the reaction shown in Scheme 1B.

**Fig. 4:**
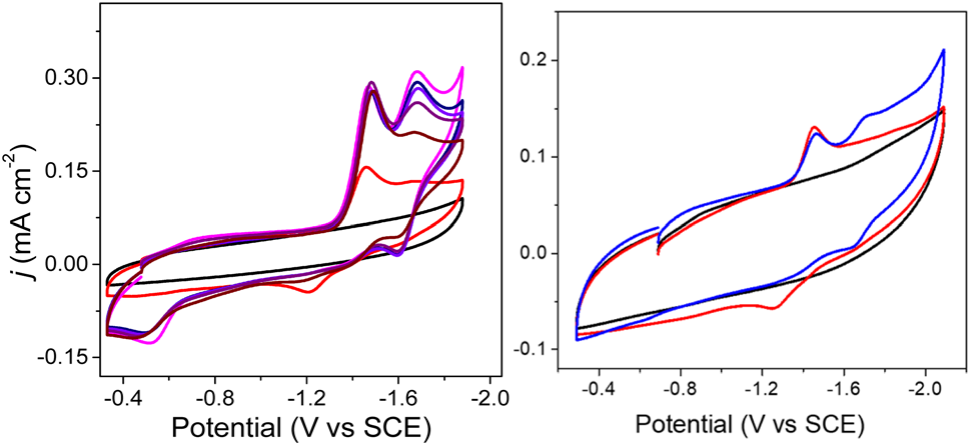
Left: cyclic voltammograms of 0.1 M Bu_4_NBF_4_ MeCN solution under 1 atm N_2_ (black), with 0.1 mM 1^−^ (red), and under 1 atm CO_2_ over 16 min where PPN_2_(1H)(1a) is formed (pink). The solution was then purged with N_2_, which initiates slow desorption of CO_2_; as seen in scans with decreased current response, recorded at 1, 6, 11, and 20 min after the N_2_ purge (other colors). Right: cyclic voltammograms under 1 atm N_2_ of 0.1 M Bu_4_NBF_4_ MeCN (black), 0.1 mM 1^−^ (red), and 0.1 mM 1^−^ with 0.11 mM benzoic acid (blue).

Cyclic voltammograms collected under CO_2_ at intervals from 1 to 18 min showed a gradual change in the cyclic voltammograms of 1^−^ over 20 min, after addition of CO_2_ and this gradual change is consistent with the knowledge that reactions of amines with CO_2_ to afford carbamate are relatively slow (Fig. 4, left).^30,31^ Based on the IR data showing no change to *ν*_CO_, we tentatively assigned the redox event at −1.43 V to overlapping reduction of both 1H and 1a^−^. When the solution of 1^−^ was purged with N_2_, the redox event at −1.67 V diminished, which is further consistent with its assignment as related to formation of 1H and 1a^−^. A differential pulse voltammetry experiment performed under 1 atm CO_2_ on a solution containing 0.1 mM Me_10_Fc and 0.1 mM 1^−^ showed two reduction events associated with 1H and 1a^−^, consistent with the cyclic voltammetry data (Fig. S11†).

The cyclic voltammogram of 1^−^ collected under 1 atm CO_2_ also shows an oxidative process at −0.5 V (Fig. 2), and this is consistent with the location where we usually observe oxidation of an intermediate catalyst hydride formed from reduction of clusters such as [Fe_4_N(CO)_12_]^–^ in the presence of protons: specifically it is equivalent to oxidation of [H–Fe_4_N(CO)_12_]^–^, and in this case it should be oxidation of (H-1H)^–^ or of (H-1a)^2−^, or an overlapping oxidation of both. The observation of a hydride oxidation event, for oxidation of (H-1H) and (H-1a)^2−^, at −0.5 V suggests that the reversible redox event with *E*_1/2_ = −1.62 V could be the redox couple associated with (H-1H)^0/–^ or (H-1a)^2−/3−^, or both. To test this hypothesis, we collected cyclic voltammograms and differential pulse voltammograms of 1^−^ under 1 atm N_2_ and in the presence of a proton source (Fig. 4 and S11†). The p*K*_a_ of 1H should be about 18.4 in MeCN, based on comparison with the known acidity of alkylamines,^32^ and so we investigated the possibility that 1H serves as a proton source for cluster-hydride formation by employing an acid that has p*K*_a_ matched to the SCS amine group. Recorded cyclic voltammograms of 0.1 mM 1^−^ in 0.1 M Bu_4_NBF_4_ MeCN under 1 atm N_2_ with 1 equivalent of BnCOOH show an irreversible reduction event at −1.43 V and a reversible wave at *E*_1/2_ = −1.62 V, Δ*E*_p_ = 130 mV (Fig. 4). In addition, an oxidation event at −0.5 V is observed. This experiment shows that the redox couple at *E*_1/2_ = −1.62 V is associated with a proton source and supports its assignment to reversible reduction of the hydride intermediates that are formed at −1.43 V.

**Scheme 2:**
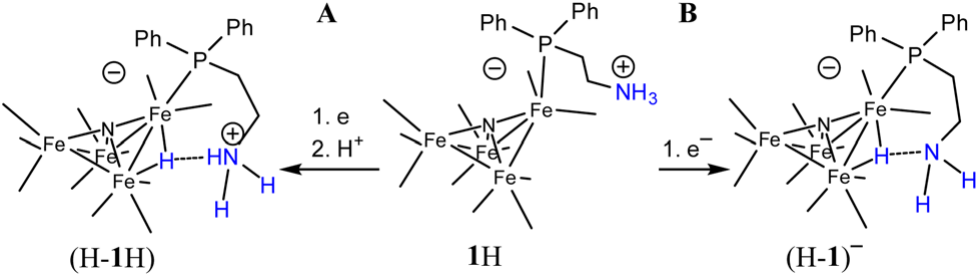
Presentation of possible H-bond stabilization that enables observation of (H-1H)^0/–^ or (H-1a)^2−^, couple at −1.63 V. (A) Proton source is from bulk solution; (B) proton source for hydride formation is the amine SCS group.

We have not previously observed reduction of [H–Fe_4_N(CO)_12_]^–^, and propose that stabilization of (H-1H) and (H-1a)^2−^ by the SCS amine (or ammonium, or carbamate) functional group is responsible for suppressing reaction of the hydride with a substrate (Scheme 2). The electron rich nature of the hydride allows for the H-bond interaction between it and the amine, or ammonium. The carbamate is less likely to interact with hydride. In prior work, a linear relationship between *E*_1/2_ and Δ*G*_Hyd_ has been established for a series of iron carbonyl clusters.^33^ We can therefore estimate the hydricities of (H-1H)^–^ and (H-2H)^–^ to be 46 and 43 kcal mol^−1^ respectively. The existence of the oxidation wave at −0.5 V even under 1 atm CO_2_, further supports the hypothesis of H-bond stabilization of hydride intermediates (H-1H) and (H-1a)^2−^, since our prior work has shown that the oxidation of [H–Fe_4_N(CO)_12_]^–^ is usually observed under N_2_ but not under CO_2_.^34^ H-bonding between hydride and an appended functional group is well-established and has been characterized in several prior cases, either crystallographically^35^ or spectroscopically.^36–38^

### Electrochemical reactivity of PPN-2 with CO_2_

**Fig. 5:**
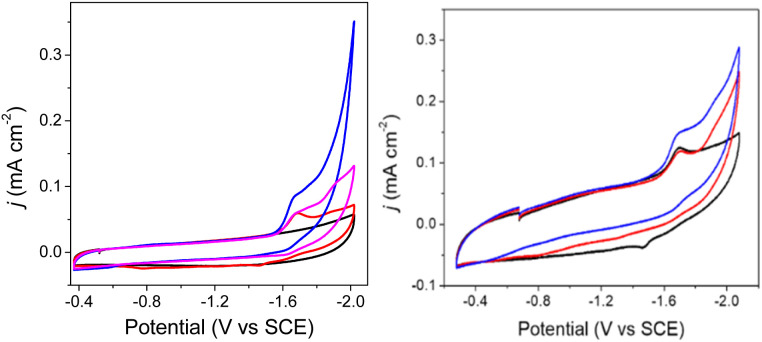
Cyclic voltammograms of 0.1 M Bu_4_NBF_4_ MeCN solutions. Left: under N_2_ (black), with 0.1 mM 2^−^ under 1 atm N_2_ (red), with 0.1 mM 2^−^ under 1 atm CO_2_ (blue), and after purging the CO_2_ saturated solution with N_2_ for 10 min, decreasing the current (pink). Right: with 0.1 mM 2^−^ under 1 atm N_2_ (black) and with added 0.5 mM ^OMe^BSulfH (red), and under 1 atm CO_2_ (blue). Blank cyclic voltammograms for 0.5 mM ^OMe^BSulfH (where 2^−^ is absent) are shown in Fig. S12.†

The *E*_½_ values for 2a^−^ are expected to be roughly equivalent to *E*_½_(2^−/2−^) = −1.67 V, since the *ν*_CO_ absorption bands for 2^−^ and 2a^−^ were observed at the same energy. Electrochemical measurements performed under 1 atm CO_2_, with solutions containing 0.1 M Bu_4_NBF_4_ MeCN and 0.1 mM 2^−^ show an increase in current density at −1.67 V, and a small reduction feature at −1.92 V, which we assign as the (H-2a)^–/2−^ couple (Fig. 2, right), consistent with our assignments made for (H-1a)^–/2−^ (Fig. 2, left). Oxidation of putative (H-2a)^–^ is also observed as a low current density feature of this cyclic voltammogram on the return oxidative scan at −0.49 V. To confirm that the features at −1.92 and −0.49 V are associated with redox chemistry of *in situ*-generated (H-2a)^–^, we performed a cyclic voltammetry experiment on solutions containing 2^−^ and ^OMe^BSulfH (Fig. 5, right). The stronger acid, BnCOOH was used in a similar study for 1^−^ (Fig. 4) but cannot be used here since H_2_ would be generated by the GC electrode at −1.92 V. The features at −1.92 and at −0.49 V were observed, but both have relatively lower current densities compared with the equivalent events associated with the redox events assigned to (H-1H) and (H-1a)^2−^ (Fig. 4, right): this suggests that stabilization of (H-2a)^–^ is not as efficient as stabilization of (H-1H) and (H-1a)^2−^. Further, this observation implies that hydride transfer (HT) from (H-1H) and/or (H-1a)^2−^ to CO_2_ should be inefficient (resulting in no or minimal formate formation), whereas HT from (H-2a)^–^ to CO_2_ might be possible.

### Electrolysis and product quantification

Controlled potential electrolysis (CPE) experiments were performed to determine whether any products of CO_2_ or proton reduction are being formed during the cyclic voltammetry experiments, and upon reductive electrolysis of 0.1 mM 1^−^ or 2^−^ under 1 atm CO_2_. The CPE experiments were followed by analysis of the head space using gas chromatography with thermal conductivity detector (GC-TCD) to quantify CO or H_2_, and analysis of the solution using high-performance liquid chromatography (HPLC) to quantify formate. We chose *p*-methoxy benzene sulfonium (^OMe^BSulfH, p*K*_a(MeCN)_ = 25.9),^39^ for the CPE experiments since it will not make H_2_ in a background reaction with the glassy carbon (GC) working electrode.

CPE experiments with 1^−^ were performed at and near the peak potential for reduction of 1^−^, at −1.40 V, with and without added 5 mM ^OMe^BSulfH, under 1 atm CO_2_. In all cases, no product resulting from a faradaic process was detected above the detection limits for CO, H_2_, or formate (Table S3 and Fig. S13–S15†). We did observe some CO formation which we attribute to cluster decomposition based on the decrease in intensity of the CO absorption bands in the IR spectrum taken after the CPE experiment (Fig. S15†). In addition, when a CPE experiment was performed with 1^−^ using ^13^CO_2_, no CO_2_ reduction products were observed in a ^13^C-NMR spectrum collected following the CPE (Fig. S16†). The CPE results obtained with 1^−^ suggest that stabilization of the (H-1)^–^ intermediate we observed in the cyclic voltammetry experiments, is preventing a HT reaction under catalytic conditions.

**Fig. 6:**
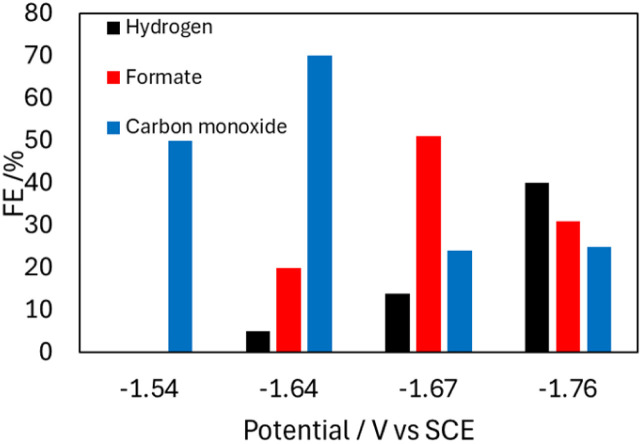
Bar graph showing the product profile relative to *E*_app_, from CPE experiments performed with 0.1 mM 2^−^ in 0.1 M Bu_4_NBF_4_ MeCN solution with 5 mM OMeBSulfH under 1 atm CO_2_ over 20 min.

CPE performed with 0.1 mM 2^−^ and 5 mM ^OMe^BSulfH, at various cathodic potentials between −1.54 and −1.74 V produced a mixture of products for which the relative amounts varied with applied potential (Fig. 6 and Table S3†). These potentials were selected based on their position near the *E*_p_. The maximum yield of formate produced was a faradaic efficiency (FE) of 51% at −1.74 V, and H_2_ and CO were also observed at this potential with FE of 14% and 24%, respectively (Calculation S1†). IR spectra recorded before and after the CPE experiment suggest that 2^−^ is 18% decomposed over the 20 min experiment and that the likely source of CO is ligands from the cluster (Fig. S15†). No CO_2_ reduced products were detected when blank CPE experiments were carried out in the absence of 2^−^ under 1 atm CO_2_. CPE experiments were also run with the used electrodes from CPE experiments containing 2^−^, and those also produced no carbon-containing products. CPE experiments carried out under 1 atm of ^13^CO_2_ atmosphere showed that formate was produced, using ^13^C-NMR spectroscopy (Fig. S17†). No ^13^CO was observed.

Taken together the results of the CPE experiments with 1^−^ and 2^−^ suggest that stabilization of the catalyst hydride by the SCS amine group is hindering HT so that both H_2_ and formate formation are suppressed, and instead some catalyst decomposition occurs, especially for 1^−^ where the HT is most hindered. The cyclic voltammetry experiments described earlier are consistent with the CPE results (Fig. 4). In particular, the suppression of H_2_ at potentials as negative as −1.67 V in the presence of organic acids is good evidence for suppression of the HT reaction. One possible reason that HT is more effectively suppressed for 1^−^ is that the reduction potential, −1.43 V, is 220 mV less reducing than for 2^−^ at −1.65 V. Another possible reason for the effective HT suppression in 1^−^ is the orientation of the primary amine functional group: in the solid-state structure of 1^−^ the amine is directed down toward the hydride active site because there is more space for the Ph rings above the cluster (Fig. 1). In the solid-state structure of 2^−^, the presence of two PPh_2_(EtNH_2_) substituents generates more steric crowding so that the amine groups (which are smaller than the Ph rings) are directed more away from the site of the hydride (Fig. 1).

### Reaction mechanism under catalytic conditions

**Scheme 3:**
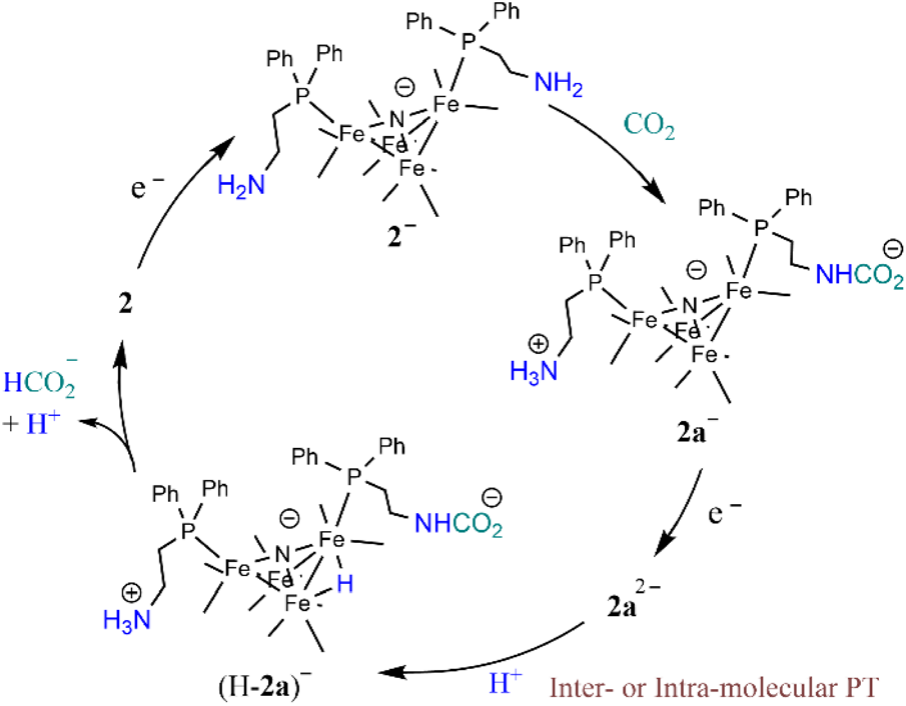
Proposed mechanism for CO_2_ reduction to formate by 2^−^.

The foregoing mechanistic investigations performed using IR spectroscopy and cyclic voltammetry, combined with the identification of products using CPE experiments, lead us to propose a mechanism for CO_2_ reduction which involves a series of chemical and ET steps including amine reactions with CO_2_ and both inter- and intra-molecular PT reactions and HT to afford formate (Scheme 3). There are some ambiguities with the proposed mechanism, which include the origin of the proton that leads to formation of (H-2a)^–^ (Scheme 3) and the origin of the CO_2_ substrate involved in HT, which could be released from the amine SCS or it could be from dissolved CO_2_ in solution. This latter question can be explored further by measuring the observed rate constant of formate formation (*k*_obs_), since this would be an approximation of the rate-determining HT step in the catalytic cycle (Scheme 3). If this rate constant is higher than we have observed for formate formation by [Fe_4_N(CO)_12_]^–^, that would suggest CO_2_ that is locally released from the SCS amine functional group might be involved in catalysis. We have previously reported *k*_obs_ for formate formation in MeCN solution from limiting current analysis as *k*_obs_ = 10 s^−1^.^17^

**Fig. 7:**
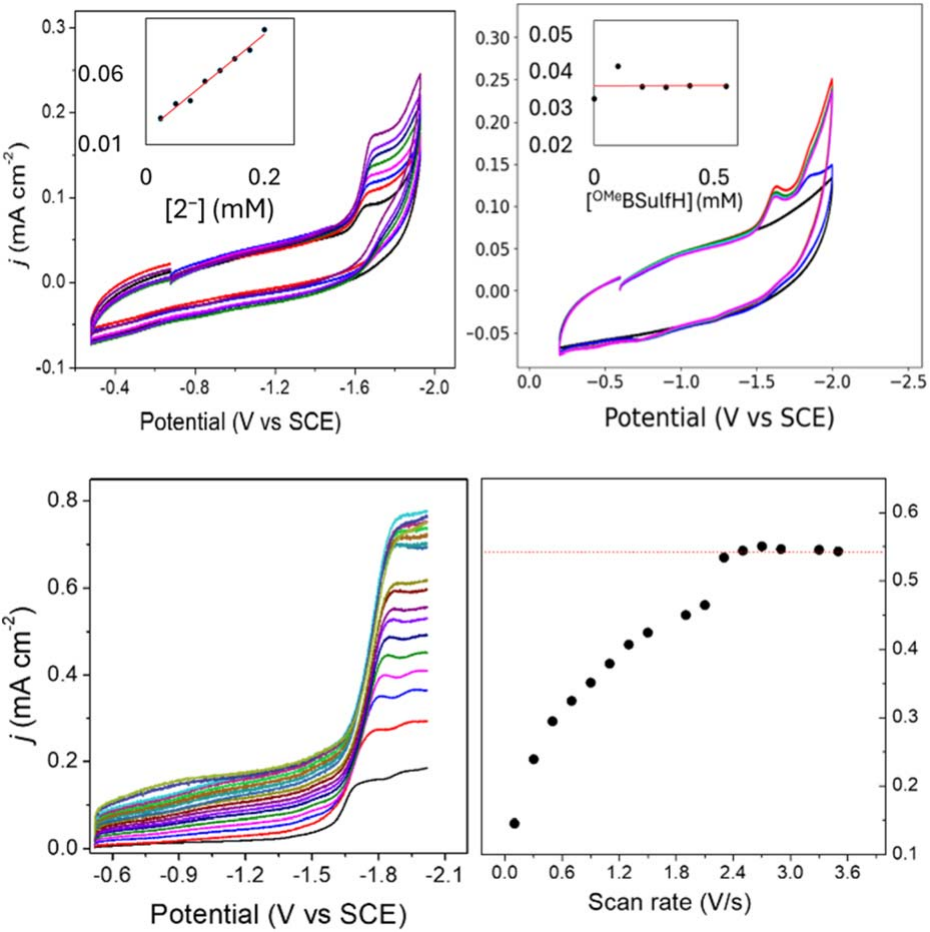
Top: cyclic voltammograms in 0.1 M Bu_4_NBF_4_ MeCN solution: (left) 1 mM ^OMe^BSulfH, with added 2^−^ at 0.025, 0. 05, 0.075, 0.1, 0.125, 0.15, 0.175, and 0.2 mM. Inset: plot of *j*_c_*vs.* [2^−^] read at −1.67 V; (right) 0.1 mM 2^−^, with added ^OMe^BSulfH. Inset: plot of *j*_c_*vs.* [^OMe^BSulfH] read at −1.67 V. GC electrode; scan rate: 100 mV s^−1^. Bottom: (left) forward cyclic voltammogram traces of 0.09 mM 2^−^ in 0.1 M Bu_4_NBF_4_ MeCN with 5 mM ^OMe^BSulfH under 1 atm CO_2_ at scan rates 0.1, 0.3, 0.5, 0.7, 0.9, 1.1, 1.3, 1.5, 1.9, 2.1, 2.3, 2.5, 2.7, 2.9, 3.3, and 3.5 V s^−1^; and (right) plot of *j*_max_*vs.* scan rate (*υ*) read at −1.95 V.

To elucidate the effect of the SCS amine functional groups on the mechanism and rate of formate production, we analysed the limiting current observed in cyclic voltammetry experiments performed with 2^−^ and an excess of CO_2_ substrate. The catalytic plateau current, *j*_c_ generated by a homogenous electrocatalyst in pure kinetic regime with excess substrate is described by eqn (1):^40,41^1
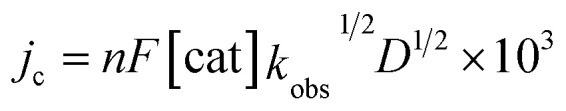
where *j*_c_ is the background-corrected plateau current density (mA cm^−2^), *n* = 2 for number of electrons for CO_2_ reduction to formate, *F* is the Faraday's constant, [cat] is [2^−^] (mol cm^−3^), *D* = 9.5 × 10^−6^ cm^2^ s^−1^ (Fig. S9†), and *k*_obs_ is the observed rate constant (s^−1^). We first measured the order of the catalytic reaction with respect to the concentration of the proton source in CPE, which is ^OMe^BSulfH, and to [2^−^]. When a solution of 0.1 mM 2^−^ in 0.1 M Bu_4_NBF_4_ MeCN under 1 atm CO_2_ was titrated with increasing amounts of ^OMe^BSulfH, the 2^−/2−^ redox couple became irreversible after 1 equivalent of ^OMe^BSulfH had been added. Addition of further ^OMe^BSulfH did not increase *j*_c_ at −1.67 V. This indicates zero-order dependence of *k*_obs_ on [^OMe^BSulfH] (Fig. 7). A solution containing 1 mM ^OMe^BSulfH under 1 atm CO_2_ was titrated with 0.025 mM up to 0.2 mM of 2^−^, and we observed that *j*_c_ increases linearly at −1.67 V, pointing toward a first-order dependence of the catalytic reaction on [2^−^] (Fig. 7). Measurement of *k*_obs_ was performed from the limiting current analysis (Calculation S2† and Fig. 7), and afforded *k*_obs_ = 22 s^−1^. A correction for the FE(HCOO^−^) was applied to give a rate constant of *k*_obs_(FE) = 7.3 s^−1^ (Calculation S3†). This rate constant is on the same order of magnitude compared to a previous measurement for the rate constant measured for formate formation by [Fe_4_N(CO)_12_]^–^. This result supports a proposal that CO_2_ substrate is derived from dissolved CO_2_ in solution. We note that 18% decomposition of 2^−^ was observed in CPE experiments performed over 20 min but the CV measurement of rate is performed over about 1 min where very little decompositions is expected.

## Conclusions

The role of SCS amine functional groups on catalyst-hydride formation and on HT reactions has been modelled in this report. Two synthetic models for an iron catalyst were prepared, containing one amine SCS group (1^−^) or two amine SCS groups (2^−^), and these amine groups were positioned near the surface active site of the catalyst with alkyl linkers. IR spectroscopy confirms the reaction of the appended amines with CO_2_ to form carbamate and ammonium. A mechanistic study, employing IR spectroscopy, cyclic voltammetry, and CPE results, determined that the SCS amine group in 1^−^ has a highly stabilizing effect on the catalyst-hydride intermediate so that formate formation is completely suppressed. The structure of 2^−^ appears to be less rigid so that stabilization of the catalyst-hydride intermediate, (H-2a)^–^, is only partially effective, according to both the cyclic voltammetry and CPE data. Formate formation by 2^−^ is therefore only partially suppressed and observed with 51% FE. The rate of formate formation by 2^−^ and [Fe_4_N(CO)_12_]^–^ are on the same order of magnitude which suggests that the substrate for catalysis by 2^−^ is not related to any behavior of the SCS carbamate functional group.

## Author contributions

The manuscript was written through contributions of all authors.

## Note added after first publication

This article replaces the version published on 13 February 2025 in which the CSD Communications https://doi.org/10.5517/ccdc.csd.cc2l8lgy and https://doi.org/10.5517/ccdc.csd.cc2l8lhz had not been cited for structures 2391540 and 2391541, respectively.

## Conflicts of interest

There are no conflicts to declare.

## Supplementary Material

SC-016-D4SC07359B-s001

## Data Availability

Crystallographic data for Et4N-1·THF and PPN-2 have been deposited at the Cambridge Crystallographic Data Centre under 2391540 and 2391541 and can be obtained from https://doi.org/10.5517/ccdc.csd.cc2l8lgy and https://doi.org/10.5517/ccdc.csd.cc2l8lhz.
