# Supplementary material for: Amine groups alter product selectivity and rate of catalytic hydride transfer reactions
Source: Chem Sci. 2025 Feb 13;16(29):13241–8. doi: 10.1039/d4sc07359b (PMC11824869; doi:10.1039/d4sc07359b)
Supplement: SC-016-D4SC07359B-s001 [file SC-016-D4SC07359B-s001.pdf]

Supplementary Information (SI) for Chemical Science.
This journal is © The Royal Society of Chemistry 2025

Supplementary Information to Accompany:

Amine Groups Alter Product Selectivity and Rate of Catalytic Hydride Transfer Reactions

Santanu Pattanayak,[†] Rachel E. Siegel,[†] Yiming Liu, James C. Fettinger, and Louise A. Berben*

Department of Chemistry, University of California, Davis, CA 95616

[†] These authors contributed equally.

email to: laberben@ucdavis.edu

Table of Contents

1. **Experimental Section**

2. **Calculations**
   Calculation S1. Calculation of Faradaic efficiency
   Calculation S2. Fast scan analysis for k_{obs} measurements
   Calculation S3. Faradaic efficiency correction of k_{obs}

3. **Tables**
   Table S1. Crystallographic data for Et_4N-**1**, PPN-**2**.
   Table S2. Selected average interatomic distances (Å) and selected average angles (deg).
   Chart S1. Atom numbering schemes for Table S2.
   Table S3. Results from CPE experiments for **1**$^-$ and **2**$^-$.

4. **Figures**
   Figures S1- S7. ^{1}H, ^{13}C and ^{31}P{^{1}H} NMR spectra and IR spectra of Et_4N-**1**, Et_4N **-2**.
   Figure S8. Differential pulse voltammograms of **1**$^-$ and **2**$^-$ under N_2
   Figure S9. Cyclic voltammograms of **1**$^-$ and **2**$^-$ at various scan rates for diffusion coefficient.
   Figure S10. Full IR spectra of the **1**$^-$ and **2**$^-$ with CO_2
   Figure S11. Differential pulse voltammograms of **1**$^-$ under N_2, CO_2, and with added benzoic acid
   Figure S12. Blank cyclic voltammograms for BnSulf under N_2 and CO_2
   Figure S13. Charge vs tie plots for CPE experiments with **1**$^-$ and **2**$^-$.
   Figure S14. Calibration curves used to quantify CPE products
   Figure S15. IR spectra of **1**$^-$ and **2**$^-$ before and after CPE
   Figure S16. ^{13}CNMR after CPE of **1**$^-$ with $^{13}CO_2$
   Figure S17. ^{13}CNMR after CPE of **2**$^-$ with $^{13}CO_2$

5. **References**

1. Experimental Section

X-ray structure determination. X-ray diffraction studies for Et_4N-**1**·2THF, and PPN-**2** were carried out on a Bruker Photon100 CMOS diffractometer, or a Bruker SMART APEX-II diffractometer equipped with a CCD detector.[1] Measurements were carried out at 90 K using Mo K_α 0.71073 Å radiation for Et_4N-**1** and PPN-**2**. The crystals were mounted on a Kaptan Loop with Paratone-N oil. Initial lattice parameters were obtained from a least-squares analysis of more than 100 centered reflections; these parameters were later refined against all data. Data collected were corrected for Lorentz and polarization effects with Saint[2] and absorption using Blessing's method and merged as incorporated with the program Sadabs.[3]

Space group assignments were based upon systematic absences, E statistics, and successful refinement of the structures. Structures were solved by direct methods with the aid of successive difference Fourier maps and were refined against all data using the SHELXT and SHELXL-2014 software package.[4] Thermal parameters for all non-hydrogen atoms were refined anisotropically. Hydrogen atoms, where added, were assigned to ideal positions and refined using a riding model with an isotropic thermal parameter 1.2 times that of the attached carbon atom (1.5 times for methyl hydrogens). Hydrogen atoms were idealized throughout the final refinement. All crystallographic calculations were performed on a Surface Pro5 with Intel i7-7660 U at 2.50 GHz with two cores, four processors and 16GB of extended memory.

Electrochemical measurements. Cyclic voltammograms were recorded under a dinitrogen (Praxair, 99.998%) atmosphere or CO_2 (Airgas) using a CH Instruments Electrochemical Analyzer Model 1400C or 620D, a glassy carbon button working electrode from BASi (surface area of 0.0707 cm^2), a platinum wire counter electrode and an $Ag/AgNO_3$ (0.001 M) reference electrode with a Vycor tip. Solutions were sparged with CO_2 for at least 20 minutes before measurements done under CO_2 to allow time for the amines to react fully. Reported potentials are all referenced to SCE couple. Ferrocene was used as an external standard where $E_{1/2}$ ferrocene/ferrocenium is 0.38 V vs. SCE in 0.1 M Bu_4NBF_4 acetonitrile (MeCN).[5] Electrolyte solution (0.1 M Bu_4NBF_4) was prepared by dissolving dry Bu_4NBF_4 in dry acetonitrile. Pure tertabutyl ammonium tetrafluoroborate for electrolyte was purified by dissolving in ethyl acetate and washing with milliQ water followed by recrystallization from boiling ethyl acetate layered with n-hexane, and dried under vacuum at 70˚C for 48 hours before use. Non-aqueous electrolyte solutions (0.1 M Bu_4NBF_4 MeCN) were stored over 3Å molecular sieves which had been activated by heating under vacuum at 200 ˚C for at least 72 hours. Differential pulse voltammograms (DPV) were recorded using Incr E (V) = 0.01, Amplitude (V) = 0.05, Pulse Width (sec) = 0.05, Sample Width (sec) = 0.0167, Pulse Period (sec) = 0.5, Quiet Time (sec) = 2 and Sensitivity (A/V) = 1e-5 parameters. All cyclic voltammograms were collected with 85% internal resistance compensation. Controlled electrode potential (CPE) experiments were performed using a Biologic multichannel (VSP 300) potentiostat.

Other Physical Measurements. After completion of a CPE experiment, 0.1 mL of solution was removed from the headspace via a Vici gastight syringe and injected into a gas chromatography-thermal conductivity detection system (GC-TCD) by Varian 3800 or Agilent 8890 using a Carboxen 1010 PLOT fused-silica column (30 m x 0.53 mm; Supelco) using N_2 (99.999%, Praxair) as the carrier gas for H_2 detection and He as the carrier gas for CO detection. The gas concentration was determined using a calibration curve prepared using varying ratios of H_2 or CO in CO_2 gas. A solution sample was removed, and formate concentration was quantified using Agilent 1260 Infinity II HPLC. All ^{1}H-NMR, ^{13}C-NMR,

and $^{31}P\{^{1}H\}$-NMR spectra were recorded at ambient temperature using a Bruker 400 MHz spectrometer, or a Bruker 600 MHz spectrometer. Chemical shifts were referenced to residual solvent. Proton decoupled phosphorus ($^{31}P\{^{1}H\}$) NMR spectra were referenced using an external H_3PO_4 standard (chemical shift of H_3PO_4 = 0 ppm). Infra-red spectra were recorded in a sealed liquid cell (SPECAC) on a Bruker Alpha Infra-red spectrometer or Bruker Invenio S. Elemental analyses were performed by the Microanalytical Laboratory at The University of California, Berkeley.

Preparation of Compounds. All manipulations were carried out using standard Schlenk or glove-box techniques under a dinitrogen (Praxair, 99.998%) atmosphere. Unless otherwise noted, dry and degassed solvents used for synthesis. Deuterated solvents were purchased from Cambridge Isotopes Laboratories, Inc., and were degassed and store over molecular sieves for overnight before use. Isotope labelled CO_2 ((13C, 99%) (< 2% 18O)) was purchased from Cambridge Isotopes Laboratories, Inc., and used without further purification. Compounds [Na(diglyme)$_2$][$Fe_4N(CO)_{12}$],[6] and diphenyl(ethyl amine)phosphine (Ph_2PEtNH_2),[7] were prepared following previously reported methods. All other reagents were purchased from commercial vendors and used without further purification.

$Et_4N[Fe_4N(CO)_{11}(Ph_2PEtNH_2)]$ (Et_4N-1). [Na(diglyme)$_2$][$Fe_4N(CO)_{12}$] (107mg, 0.124 mmol) and 1 eq. of Ph_2PEtNH_2 (38.4 mg, 0.124 mmol) were dissolved in 3 mL THF in a vial sealed with a Teflon lined screw cap. The reaction mixture was heated at 60 °C for 16 h. After it was cooled, the reaction mixture was filtered over a 2 cm Celite plug, and an equal volume of hexane was added so that a black precipitate formed which is a Na(diglyme)$_x$(THF)$_y$ salt of **1**$^-$, x and y are unknown. The black powder (96.4 mg) was collected by filtration and then used directly in a salt metathesis reaction with Et_4NCl, without further purification or characterization. A mixture of the black powder (96.4 mg) and Et_4NCl (15.3mg, 0.092 mmol) were stirred in 5 mL of THF for 30 min before the THF was pulled off and 2 mL CH_2Cl_2 was added to the residue which was stirred for 10 min. The CH_2Cl_2 solution was then filtered through a celite plug followed by removal of CH_2Cl_2 in vacuum. A black crystalline powder (72.6 mg, 88 % yield) was obtained from a concentrated THF solution of Et_4N-**1** layered with hexane solution held at −16 °C for about 3 days. IR ν_{CO}(MeCN): 2037 (s), 1985 (vs), 1970 (vs), 1964 (sh), 1931 (w) cm^{-1}. Combustion analysis calculated for $C_{37}H_{40}Fe_4N_2O_{12}P$: C, 45.07; H, 3.58; N, 2.84. Found: C, 45.03; H, 3.66; N, 2.73. ^{1}H NMR (400 MHz, MeCN-d_3) δ 7.69 (dtd, J = 9.7, 5.2, 4.7, 3.2 Hz, 4H, Ar), 7.50 – 7.35 (*m,* 6H, Ar), 3.70 – 3.59 (*m*, 2H, THF), 3.15 (q, J = 7.3 Hz, 8H, NCH_2CH_3), 2.98 (m, 2H, $Ph_2PCH_2CH_2NH_2$), 2.63 – 2.51 (m, 2H, $Ph_2PCH_2CH_2NH_2$), 1.84 – 1.76 (m, 2H, THF), 1.21 (ddt, J = 7.2, 3.7, 1.8 Hz, 12H, NCH_2CH_3). ^{13}C NMR (151 MHz, CD_3CN) δ 220.31 (d, J = 12.5 Hz, CO), 218.53 (s, CO), 215.88 (s, CO), 136.81 (d, J = 40.2 Hz, Ar), 133.06 (d, J = 10.1 Hz, Ar), 130.83 (d, J = 1.9 Hz, Ar), 129.27 (d, J = 9.7 Hz, Ar), 68.24 (s, THF), 56.35 – 49.39 (m, NCH_2CH_3), 39.34 (s, $Ph_2PCH_2CH_2NH_2$), 35.39 (s, $Ph_2PCH_2CH_2NH_2$), 26.20 (s, THF), 7.61(m, NCH_2CH_3). $^{31}P\{^{1}H\}$ NMR (162 MHz, CD_3CN) δ 52.52 ppm. IR ν_{CO}(MeCN): 2037 (s), 1985 (vs), 1970 (vs), 1964 (sh), 1931 (w) cm^{-1}. The PPN-**1** salt could also be obtained by performing a salt metathesis reaction with the unpurified Na-**1** and PPNCl to afford PPN-**1**, followed by crystallization from a THF solution layered with hexane and held at −16 °C for about 4 days.

$Et_4N[Fe_4N(CO)_{10}(Ph_2PEtNH_2)_2]$ (Et_4N-**2**). [Na(diglyme)$_2$][$Fe_4N(CO)_{12}$] (148 mg, 0.171 mmol) and 3.5 eq. of Ph_2PEtNH_2 (54.1 mg, 0.171 mmol) were dissolved in 1 mL THF and 3 mL Toluene in a vial sealed with a Teflon lined screw cap. The reaction mixture was heated at 80 °C for 24 hours and then filtered through a 2 cm Celite plug. The resulting solid was washed with diethyl ether to afford 184.7 mg

(68%, 0.16 mmol) black powder which is a Na salt of **2**⁻. Reaction of the black powder with Et_4NCl (29.2 mg, 0.18 mmol) in CH_2Cl_2 was performed over 2 h, and the reaction solution was filtered through a small Celite plug. The CH_2Cl_2 was removed under vacuum and the resulting black powder was washed three times with Et_2O (3 × 4 mL) and dried *in vacuo* to afford Et_4N-**2** (107 mg, 63 % yield). A black crystalline powder (72.6 mg, 69 % yield) was obtained from a concentrated THF solution of Et_4N-**2** layered with hexane solution held at -16 °C for about 8 days. ^1H NMR (400 MHz, MeCN-d_3) δ 7.77 – 7.54 (m, 8H, Ar), 7.36 (dt, J = 4.7, 1.9 Hz, 12H, Ar), 3.65 (ddd, J = 6.6, 3.4, 1.7 Hz, 2H, THF), 3.15 (q, J = 7.3 Hz, 8H, NCH_2CH_3), 2.69 (s, 4H, $Ph_2PCH_2CH_2NH_2$), 2.56 – 2.39 (m, 4H, $Ph_2PCH_2CH_2NH_2$), 1.85 – 1.70 (m, 2H, THF), 1.25 – 1.17 (m, 12H, NCH_2CH_3). ^{13}C NMR (151 MHz, CD_3CN) δ 221.40 (d, J = 11.2 Hz, CO), 220.28 (s, CO), 137.93 (d, J = 37.0 Hz, Ar), 133.40 (d, J = 10.0 Hz, Ar), 130.21 (s, Ar), 128.89 (d, J = 9.2 Hz, Ar), 68.23 (s, THF), 53.40 – 52.61 (m, NCH_2CH_3), 39.17 (s, $Ph_2PCH_2CH_2NH_2$), 37.33 (s, $Ph_2PCH_2CH_2NH_2$), 26.19 (s, THF), 7.61 (m, NCH_2CH_3). $^{31}P\{^1H\}$ NMR (162 MHz, CD_3CN) δ 49.22 ppm. IR v_{CO}(MeCN): 2006 (s), 1959 (vs) 1943 (s), 1932 (sh), 1914 (w) cm^{-1}. Combustion analysis calculated for $C_{39}H_{32}Fe_4N_3O_{10}P_2$: C, 44.80; H, 3.25; N, 2.82. Found: C, 44.23; H, 3.52; N, 2.83. We were unable to grow crystals of Et_4N-**2** that are suitable for X-ray diffraction studies, and so a salt metathesis reaction with the unpurified Na-**2** and PPNCl was performed to afford PPN-**2**, and this was crystallized from a THF solution layered with hexane and held at −16 °C to obtain black block-shaped crystals of PPN-**2**.

2. Calculations

Calculation S1. Calculation of Faradaic efficiency

The theoretical yield of formate can be calculated using the charge passed during electrolysis:

$$\text{yield} = \frac{Q}{nF}$$

Where Q is the amount of charge passed during electrolysis (C), n is the number of electrons involved in catalysis (2), and F is faraday's constant (C mol^{-1}).

This theoretical yield can then be used to determine the faradaic efficiency:

$$FE = \frac{mols\ formate\ (experimental)}{mols\ formate\ (theoretical)} \times 100\ \%$$

Calculation S2. Fast scan method for measurement of observed rate (k_{obs}/s^{-1}).

A fast scan method introduced by Azcarate, I. et al[8] and Cometto, C. et al,[9] was used to calculate the observed rate constant (k_{obs}) for CO_2 reduction to formate by **1**$^-$. In fast scan method applying a very high scan rate results a scan rate independent region (pure kinetic regime) due to mutual compensation of catalyst diffusion and observed rate of catalysis.
The catalytic plateau current, j_c generated by a homogenous electrocatalyst at pure kinetic regime is defined as equation S1[10]:

$$j_c = nF[cat]k_{obs}^{1/2}D^{1/2} \times 10^3 \qquad \text{(Equation S1)}$$

where, j_c is the background corrected plateau current density (mA cm^{-2}) at the scanning potential E, $n = 2$ for number of electrons for CO_2 reduction to formate, [Cat] is [**2**$^-$] in mol cm^{-3}, k_{obs} is the observed rate constant (s^{-1}) and all other variables have been defined previously.
Parameters used to calculate the observed rate constant are:

$j_c = 0.54$ mA cm^{-2}, [**2**$^-$] is 9×10^{-8} mol cm^{-3}, $k_{obs} = 22.4$

Calculation S3. Faradaic efficiency correction of k_{obs}

A method for corrected observed rates based on faradaic efficiency has been previously reported by Robert and coworkers according to equation S2[11]:

$$k_{\text{obs, corrected}} = k_{\text{obs}} \times \left(\frac{FE}{100}\right)^2$$

The k_{obs} values obtained were corrected for the measured FE which is 51 %. Therefore, the value for k_{obs} is 7.3 s^{-1}

3. Tables

Table S1. Crystal data and structure refinement for $Et_4N[Fe_4N(CO)_{11}(Ph_2PEtNH_2)]$ (Et_4N-**1**·2THF); and $PPN[Fe_4N(CO)_{10}(Ph_2PEtNH_2)_2]$ (PPN-**2**).

	Et_4N-**1**·2THF	PPN-**2**
Formula	$C_{41}H_{52}Fe_4N_3O_{13}P$	$C_{74}H_{62}Fe_4N_4O_{10}P_4$
Crystal size /mm^3	0.379 × 0.266 × 0.173	0.832 × 0.721 × 0.318
Formula wt, gmol^{-1}	1049.22	1514.55
Space group	$P2_1/c$	$P\bar{1}$
a, Å	17.157(4)	12.2009(6)
b, Å	10.6828(9)	16.4108(8)
c, Å	25.135(2)	17.7080(8)
α, deg	90	82.274(2)
β, deg	97.4945(14)°.	76.135(3)
γ, deg	90	84.742(3)°.
V, Å^3	4567.5(12)	3404.5(3)
Z	4	2
T, K	90(2)	90(2)
ρ, calcd, g cm^{-3}	1.728	1.477
Refl. collected/2θ_{max}	32541/50.484	26673/50.484
Unique refl./ $I > 2\sigma(I)$	8422/5990	15724/13089
No. param/restrains	767/0	1113/4
λ, A°/μ (Kα), cm^{-1}	0.71073	0.71073
R_1/GOF	0.0386/1.013	0.0332/1.021
wR_2 ($I > 2\sigma(I)$) ^{a}	0.0740	0.0804
Res. density, e Å^{-3}	0.419/-0.419	0.541/−0.434

Table S2. Selected bond lengths and angels for $Et_4N[Fe_4N(CO)_{11}(Ph_2PEtNH_2)]$ (Et_4N-**1**·2THF); and $PPN[Fe_4N(CO)_{10}(Ph_2PEtNH_2)_2]$ (PPN-**2**)

	Et_4N-**1**·2THF		PPN-**2**
Fe_1-N	1.771(3)	Fe_1-N	1.7857(16)
Fe_2-N	1.915(3)	Fe_2-N	1.7922(16)
Fe_3-N	1.780(3)	Fe_3-N	1.9235(15)
Fe_4-N	1.907(3)	Fe_4-N	1.9270(15)
Fe_1-Fe_2	2.5920(7)	Fe_1-Fe_3	2.5857(4)
Fe_1-Fe_4	2.6426(7)	Fe_1-Fe_4	2.6204(4)
Fe_2-Fe_3	2.6086(7)	Fe_2-Fe_3	2.6159(4)
Fe_2-Fe_4	2.4959(7)	Fe_2-Fe_4	2.5848(4)
Fe_3-Fe_4	2.5935(7)	Fe_3-Fe_4	2.5078(4)
Fe_1-P	2.2066(10)	Fe_1-P	2.2102(5)
Fe3-P	--	Fe_2-P	2.207(6)
<Fe_4-Fe_2-Fe_3>	61.024(18)	<Fe_3-Fe_4-Fe_2>	61.796(11)
<Fe_3-Fe_4-Fe_1>	85.37(2)	<Fe_2-Fe_4-Fe_1>	86.804(12)
<Fe_1-Fe_2-Fe_3>	86.10(2)	<Fe_1-Fe_3-Fe_2>	86.879(12)
<Fe_1-N_1-Fe_3>	178.41(16)	<Fe_1-N_1-Fe_2>	176.98(10)

Chart S1. Numbering scheme used in Table S2

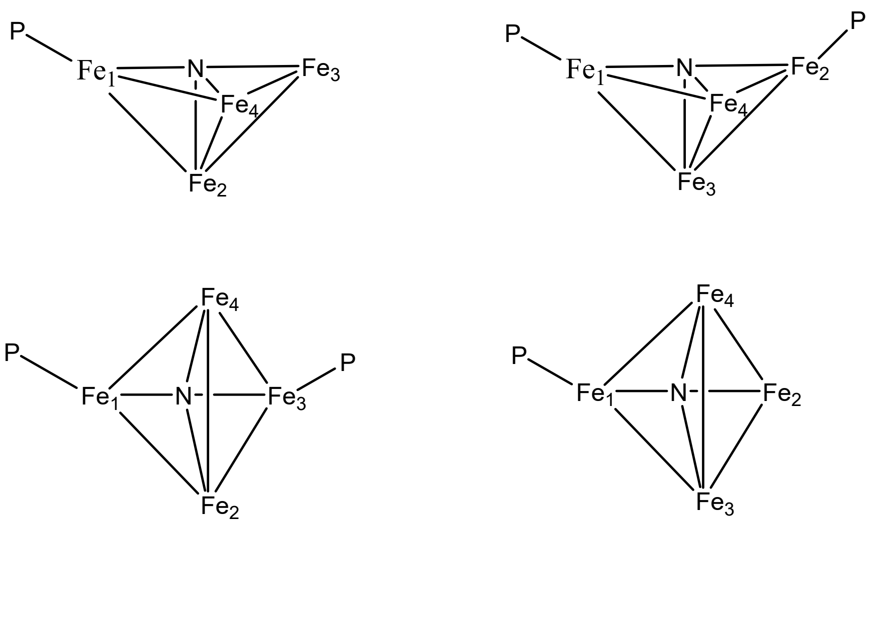

Table S3. Results from CPE experiments under N_2 and CO_2 with 0.1 mM **1**$^{-}$ and **2**$^{-}$ in 0.1 M Bu_4NBF_4 MeCN solution over 20 min.

	E	*q* /C	FE /% HCO_2^-	FE /% H_2	FE /% CO
**1**$^{-}$	−1.3	1.2	nd	nd	200
	−1.4	6.2	nd	nd	80
**2**$^{-}$	−1.54 [a]	2.1	nd	nd	50
	−1.64 [a]	4.2	20	5	70
	−1.67 [a]	5.6	51	14	24
	−1.76 [a]	6.8	31	40	25
None	−1.67	1.1	nd	28	nd

[a] with added 5 mM ^{OMe}BSulfH.

4. Figures

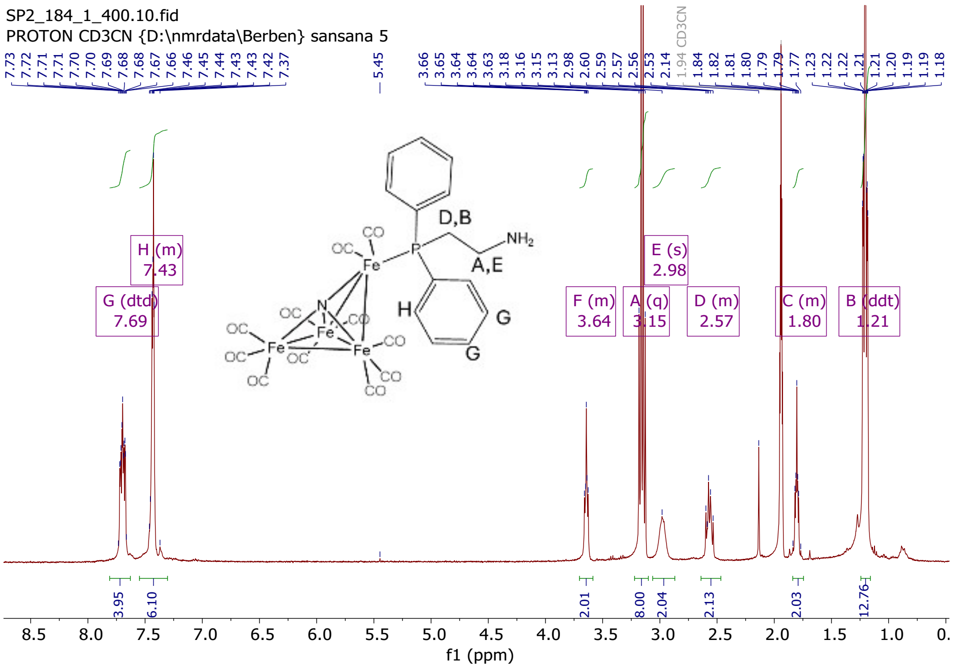

Figure S1. ^{1}H NMR spectrum (CD_3CN, 298 K, 400 MHz) of Et_4N-**1**. Peaks F and C are from THF.

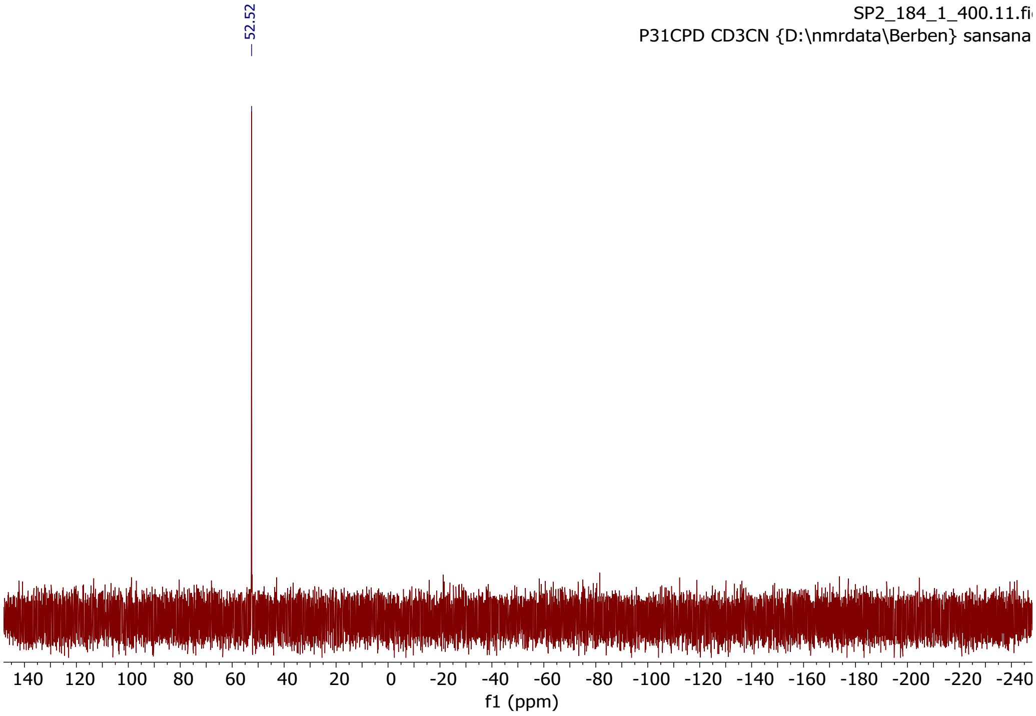

Figure S2. $^{31}P\{^{1}H\}$ NMR spectrum (CD_3CN, 298 K, 162 MHz) of Et_4N-**1.**

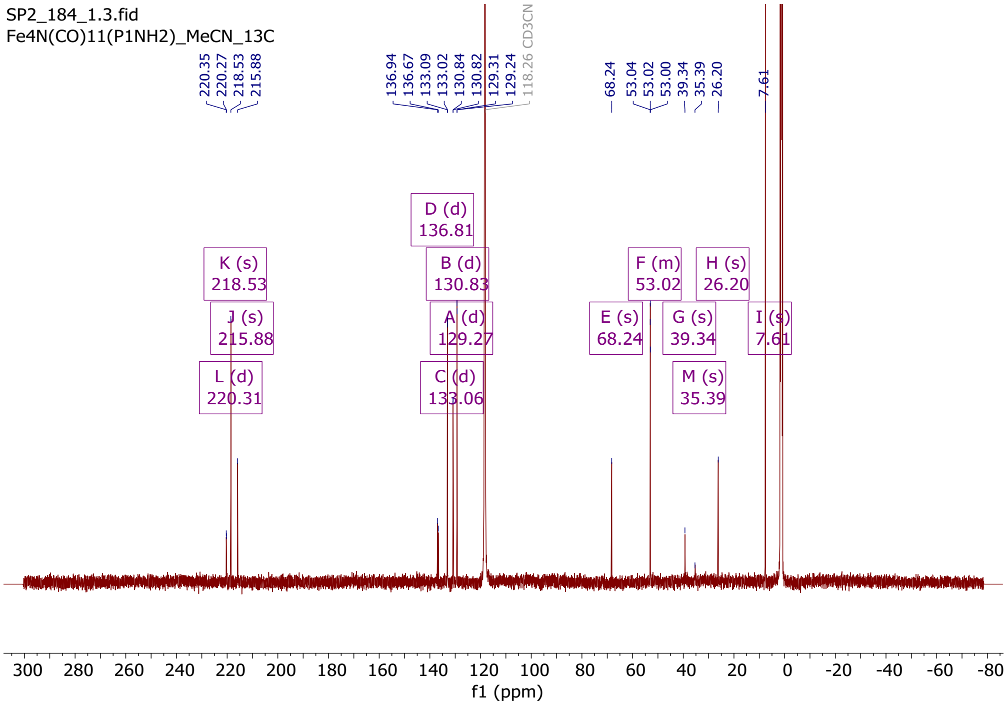

Figure S3. $^{13}C\{^{1}H\}$ NMR spectrum (CD_3CN, 298 K, 151 MHz) of Et_4N-**1**.

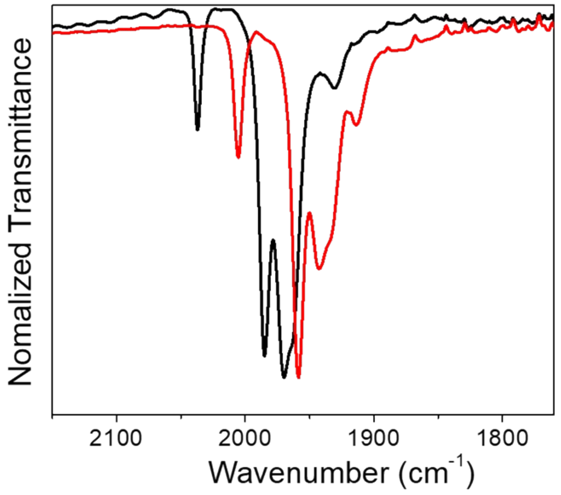

Figure S4. Plot of normalized IR data of PPN–**1** (black) and PPN–**2** (red) in MeCN under N_2.

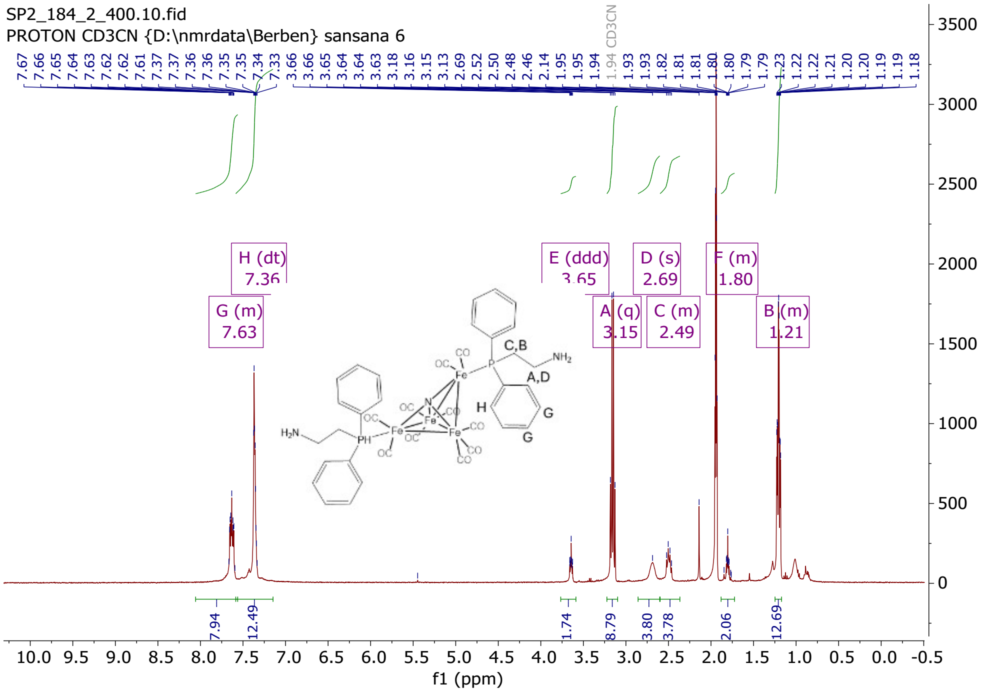

Figure S5. ^{1}H NMR spectrum (CD3CN, 298K, 400 MHz) of Et_4N-**2**. Peaks E and F are from THF

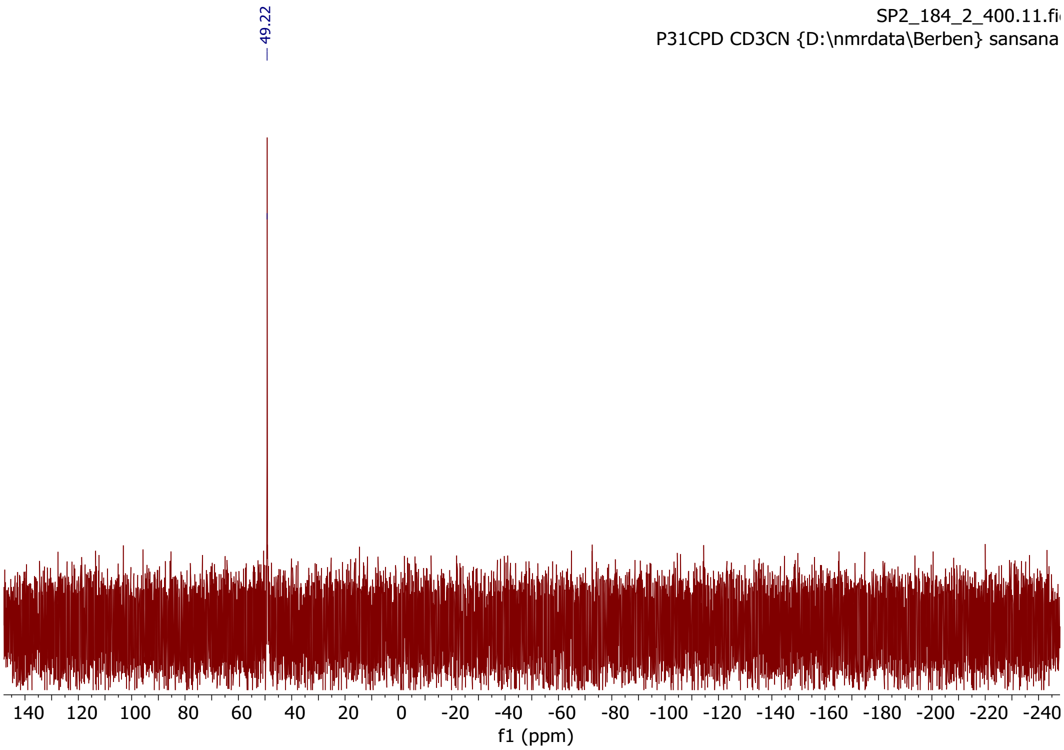

Figure S6. ^{31}P{^{1}H} NMR spectrum (CD_3CN, 298K, 162 MHz) of Et_4N-**2**.

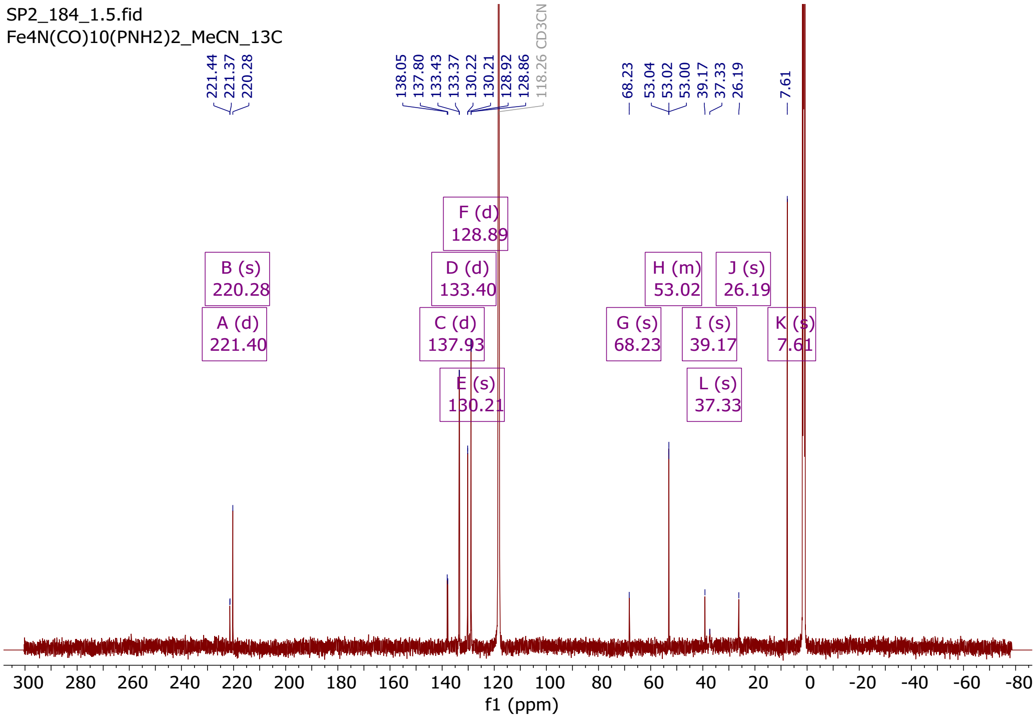

Figure S7. $^{13}C\{^1H\}$ NMR spectrum (CD_3CN, 298K, 151 MHz) of Et_4N-**2.**

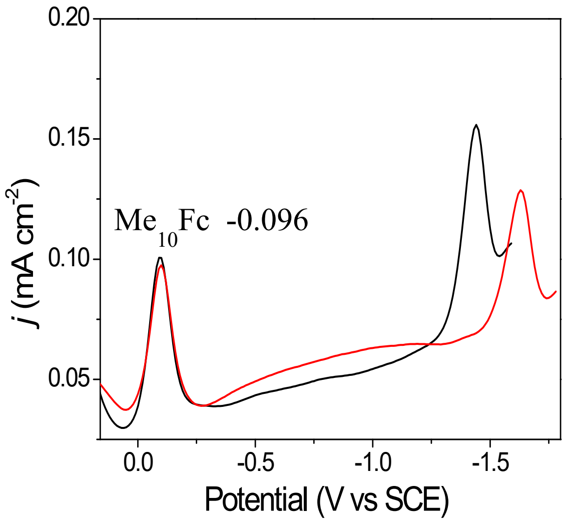

Figure S8. Differential pulse voltammograms of 0.1 mM $\mathbf{1}^-$ (black) and $\mathbf{2}^-$ (red) in 0.1 M Bu_4NBF_4 MeCN under 1 atm N_2 in presence of 0.1 mM decamethyl ferrocene ($Me_{10}Fc$). Redox potential for $Me_{10}Fc$ was set to -0.096 V vs SCE.[ref] A comparison of area under the curve for $\mathbf{1}^-$ (black) and $\mathbf{2}^-$ (red) at -1.43 and -1.65 V vs SCE respectively, with the peak area for $Me_{10}Fc$ at -0.096 is almost same which resembles 1e-transfer associated with each redox event.

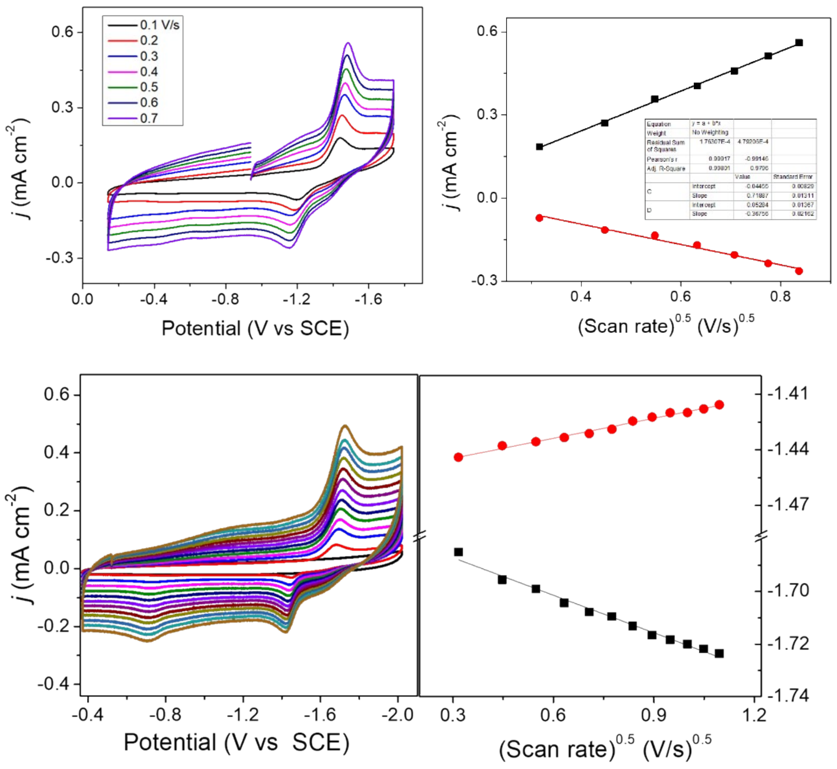

Figure S9. (top) cyclic voltammograms of **1**$^-$, at scan rates shown in the legend; and (bottom) cyclic voltammograms of **2**$^-$ (scan rates 0.1. 0.2, 0.3, 0.4, 0.5,0.6, 0.7,0.8, 0.9, 1, 1.1, 1.2 V^{s-1}).

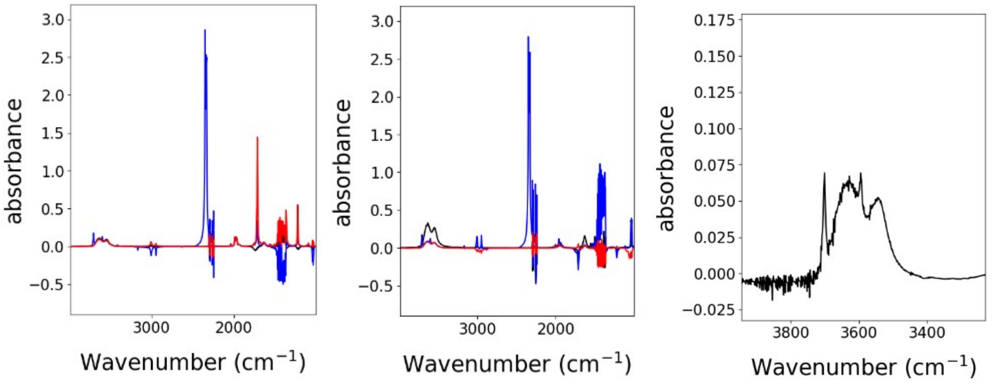

Figure S10. IR spectrum of: 0.7 mM **1**$^-$ (left) and **2**$^-$ (middle); both in MeCN, under 1 atm of N_2 (black), under 1 atm CO_2 (blue), and with 0.7 mM of benzoic acid. (right): 5 mM commercial dimethylammonium dimethylcarbamate in MeCN, under 1 atm of N_2. Benzoic acid used in this experiment has an IR peak in the same region as carbamate (~1700 cm^{-1}). Therefore, the high energy bands at 3700 and 3594 cm^{-1} serve as the best reference for carbamate formation under these conditions.

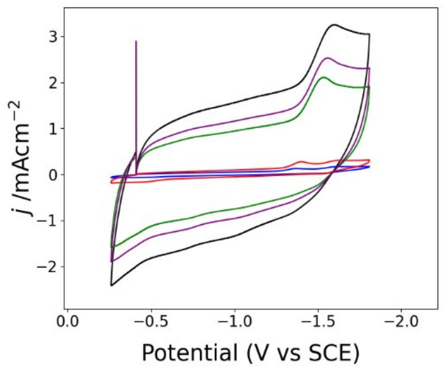

Figure S11. Cyclic voltammograms of 0.05 mM **1**⁻ under 1 atm CO_2 (scan rates 0.1, 0.5, 15, 20, 30 V^{s-1}).

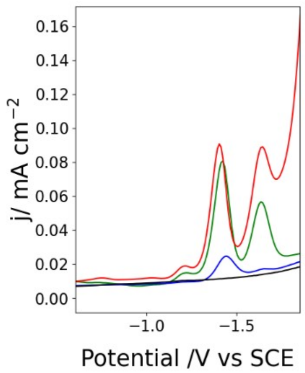

Figure S11. Differential pulse voltammograms of 0.1 M Bu_4NBF_4 MeCN solutions: (black) under 1 atm N_2, (blue) with 0.1 mM **1**⁻ 1 atm N_2, (red) with 0.1 mM **1**⁻ and 0.11 mM benzoic acid under N_2, and (green) with 0.1 mM **1**⁻ under CO_2. The increase in current under 1 atm CO_2 is attributed to the electrocatalytic reduction of CO_2.

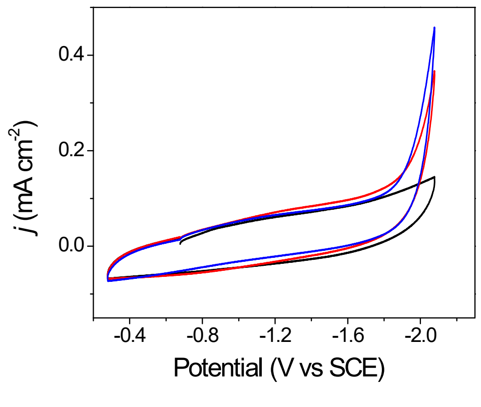

Figure S12: Cyclic voltammograms of 0.1 M Bu_4NBF_4 MeCN under 1 atm N_2 (black), with 2 mM ^{OMe}BSulfH under 1 atm N_2 atmosphere (red), and with 2 mM ^{OMe}BSulfH under 1 atm CO_2 atmosphere (blue).

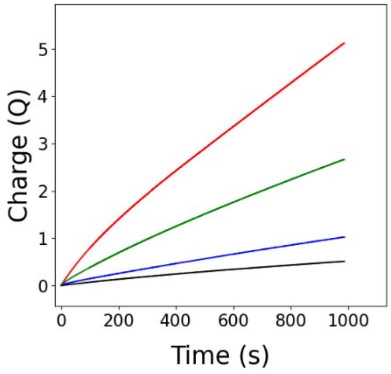

Figure S13: Charge vs time plots for electrolysis in 0.1 M Bu_4NBF_4 under 1 atm CO_2 with: no added catalyst (black), 0.1 mM **1**$^-$ (blue), 0.1 mM **2**$^-$ and ^{OMe}BSulfH (red), and with a used, rinsed electrode in fresh solution (green).

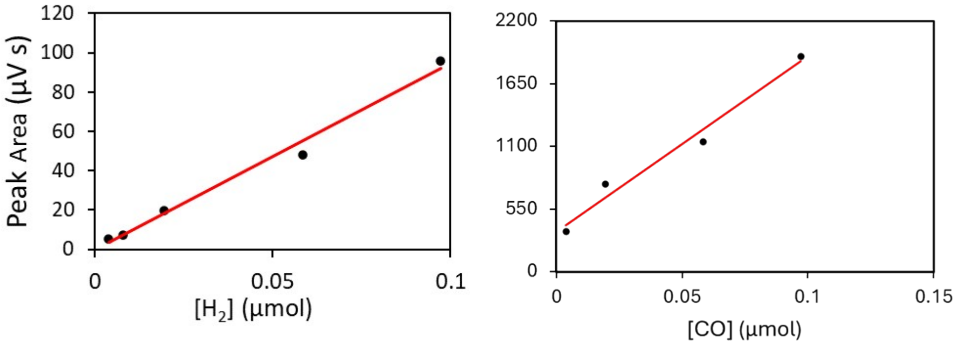

Figure S14: (left) Calibration curve used to quantify H_2 yields from GC-TCD data. The plot was made with samples made by dilution of H_2 in CO_2. (right) Calibration curve used to quantify CO yields from GC-TCD data. The plot was made with samples made by dilution of CO in CO_2.

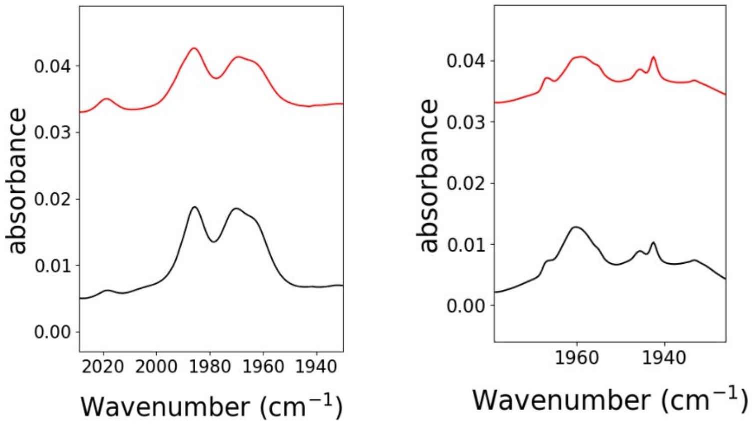

Figure S15. (left) IR spectra of 0.1 mM **1**$^-$ in 0.1 M Bu_4NBF_4 MeCN CPE solution before (black line) and after (red line) the electrolysis. Decrease in absorption corresponds to ~33% decomposition during electrolysis. (right) IR spectra of 0.1 mM **2**$^-$ in 0.1 M Bu_4NBF_4 MeCN CPE solution before (black line) and after (red line) the electrolysis.

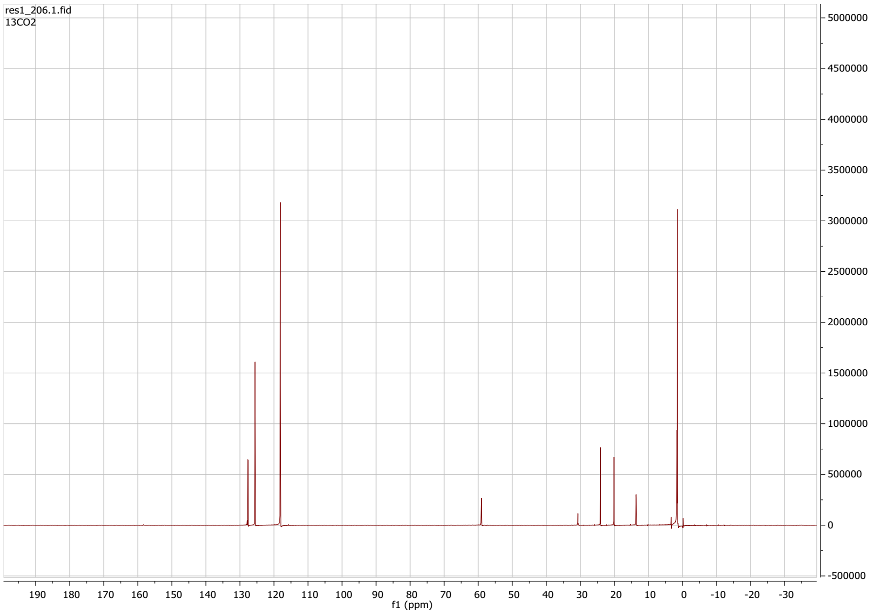

Figure S16. $^{13}C\{^{1}H\}$ NMR of electrolyte solution after CPE with 0.1 mM **1**$^{-}$ under 1 atm $^{13}CO_2$. C_6D_6 is observed at 128.1 ppm. Peaks at 117.6, 58.6, 23.6, 22.0, 19.6 and 0.99 ppm correspond to acetonitrile and tertabutyl ammonium cation from electrolyte solution. CO (184 ppm) was not observed.

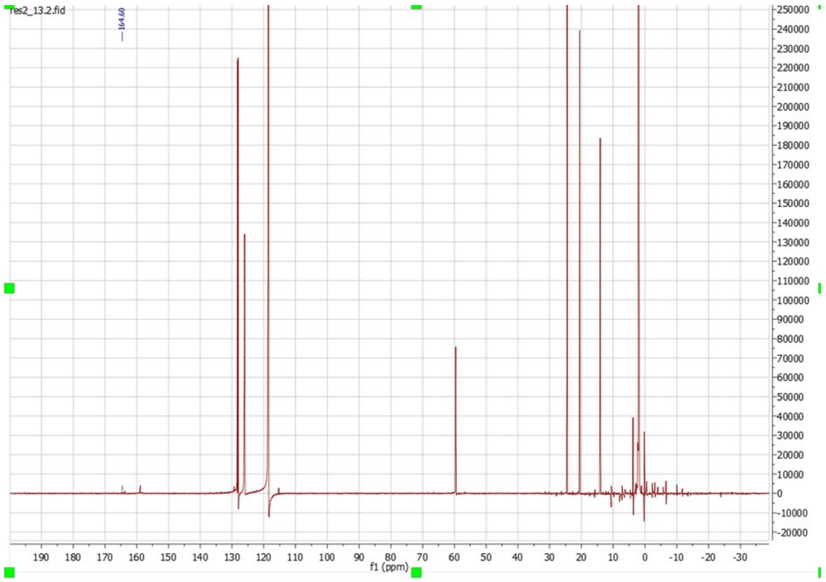

Figure S17. ^{13}C-NMR spectrum collected after CPE experiment with 0.1 mM **2**$^{-}$ under 1 atm $^{13}CO_2$. Peak at 164.6 ppm is consistent with formic acid which indicates that $^{13}CO_2$ has been converted into $H^{13}COOH$. Formate and formic acid likely exist in an equilibrium due to the pK_a of formic acid (20.9)[12] and the estimated pK_a of the appended ammonium (18.4).

5. References

[1] SMART Software Users Guide, Version 5.1, Bruker Analytical X-Ray Systems, Inc., Madison, WI, 1999.

[2] Bruker (**2019**) APEX3 (Version 2019.1) and (**2016**) SAINT (Version 8.37a). Bruker AXS Inc., Madison, Wisconsin, USA.

[3] (a) Blessing, R. H. An Empirical Correction for Absorption Anisotropy. *Acta Crystallogr., Sect. A: Found. Adv* **1995**, *51*, 33-38; (b) Sheldrick, G.M., SADABS (**2016**) Version 2016/2, 'Siemens Area Detector Absorption Correction' Universität Göttingen: Göttingen, Germany.

[4] (a) Sheldrick, G. M. (**2014**) SHELXT, Universität Göttingen: Göttingen, Germany. Structure determination program. Private communication; (b) Sheldrick, G. M. (**2017**). SHELXL2017/1. Universität Göttingen: Göttingen, Germany.

[5] House, H. O.; Feng, E.; Peet, N. P. A Comparison of Various Tetraalkylammonium Salts as Supporting Electrolytes in Organic Electrochemical Reactions. *J. Org. Chem.* **1970**, *36*, 2371–2375.

[6] (a) Rail, M. D.; Berben, L. A. Directing the Reactivity of $[HFe_4N(CO)_{12}]^-$ Toward H^+ or CO_2 Reduction by Understanding the Electrocatalytic Mechanism. *J. Am. Chem. Soc.* **2011**, *133*, 18577–18579; (b) Nguyen, A. D.; Rail, M. D.; Shanmugam, M.; Fettinger, J. C.; Berben, L. A. Electrocatalytic Hydrogen Evolution from Water by a Series of Iron Carbonyl Clusters. *Inorg. Chem.* **2013**, *52*, 12847-12854.

[7] T. Hayashi, M. Konishi, M. Fukushima, K. Kanehira, T. Hioki and M. Kumada, J. Org. Chem., 1983, 48, 2195.

[8] Azcarate, I.; Costentin, C.; Robert, M.; Savéant, J. M. Through-Space Charge Interaction Substituent Effects in Molecular Catalysis Leading to the Design of the Most Efficient Catalyst of CO_2-to-CO Electrochemical Conversion. *J. Am. Chem. Soc.* **2016**, *138*, 16639–16644.

[9] Cometto, C.; Chen, L.; Anxolabéhère-Mallart, E.; Fave, C.; Lau, T.-C.; Robert, M. Molecular Electrochemical Catalysis of the CO_2-to-CO Conversion with a Co Complex: A Cyclic Voltammetry Mechanistic Investigation. *Organometallics* **2019**, *38*, 1280– 1285.

[10] Savéant J. -M., *Elements of Molecular and Biomolecular Electrochemistry*, John Wiley & Sons, Hoboken, **2006**.

[11] Cometto, C.; Chen, L.; Anxolabéhè-Mallart, E.; Fave, C.; Lau,T.-C.; Robert, M. Molecular Electrochemical Catalysis of the CO_2-to-CO Conversion with a Co Complex: A Cyclic Voltammetry Mechanistic Investigation. *Organometallics*, **2019**, *38*, 1280−1285.

[12] Stirling, M. J.; Sweeney, G; MacRory, K.; Blacker, A.J.; Page M.I. The kinetics and mechanism of the organo-iridium-catalysed enantioselective reduction of imines. *Org Biomol Chem*. **2016**, *14*, 3614–3622.
